# Molecular and Clinicopathological Biomarkers Predicting Brain Metastasis in Triple-Negative Breast Cancer: A Systematic Review

**DOI:** 10.3390/ijms27041909

**Published:** 2026-02-16

**Authors:** Savi Agarwal, Pasha Mehranpour, Anjani Chawla, Carissa Vaish, Simon Han, Isaac Yang, Madhuri Wadehra

**Affiliations:** 1 Department of Neurosurgery, University of California, Los Angeles, CA 90095, USA; saviagarwal@mednet.ucla.edu (S.A.); simonhan@mednet.ucla.edu (S.H.); iyang@mednet.ucla.edu (I.Y.); 2 School of Medicine, California University of Science and Medicine, 1501 Violet Street, Colton, CA 92324, USA; pasha.mehranpour@md.cusm.edu; 3 Department of Biochemistry and Biomedical Sciences, McMaster University, Hamilton, ON L8S 4L8, Canada; chawla23@mcmaster.ca; 4 School of Public Health, University of Washington, Seattle, WA 98195, USA; cvaish@uw.edu; 5 Department of Pathology and Laboratory Medicine, University of California, Los Angeles, CA 90095, USA

**Keywords:** triple-negative breast cancer, brain metastasis, predictive biomarkers, metastatic mechanisms, prognostic indicators

## Abstract

Almost half of patients with triple-negative breast cancer (TNBC) develop brain metastasis (TNBCBM), a marker of poor prognosis. TNBC is a more aggressive breast cancer subtype which lacks ER, PR, and HER2 expression, and thus, exploring predictive biomarkers is crucial to improving TNBCBM outcomes through targeted therapy. To curate these biomarkers, peer-reviewed publications from 2010 to 2025 were extracted from PubMed, Scopus, Embase, Cochrane, and Web of Science if they evaluated clinicopathological biomarkers of TNBCBM. A total of 130 studies (60 clinical and 70 pre-clinical) were included. Publications most often featured transcriptomic studies, growth factor receptors, and immune microenvironment markers with 37, 19, and 17 studies identified, respectively. While TNBC aggressiveness has been linked to metastasis, advancing stage, and poor prognosis, several studies focused on utilizing circulating protein and transcriptomic biomarkers for early detection. While few pathways appeared specifically for TNBCBM, investigating these biomarkers further may allow for improved risk stratification, clinical trial design, patient selection, and therapeutic development. Identification of the most promising biomarkers will pave the way for improved prognosis of the most lethal complications of TNBC.

## 1. Introduction

Metastatic lesions account for about 15% of all central nervous system (CNS) tumors, with lung cancer being the most common source (35%), followed by breast cancer (30%). Of the breast cancer subtypes, triple-negative breast cancer (TNBC) is the most common subtype to metastasize to the CNS [1]. TNBC is a particularly aggressive form of breast cancer, defined by the lack of expression of estrogen receptor (ER), progesterone receptor (PR), and human epidermal growth factor receptor 2 (HER2) [2,3]. The lack of these molecular features posed clinical challenges in treatment, as established therapies that target receptors, such as hormonal interventions or trastuzumab, were rendered ineffective. Patients with TNBC therefore experience higher rates of recurrence, metastasis, and mortality compared to other forms of breast cancer, with half of all primary TNBC patients remaining susceptible to brain metastasis (BM) even after resection and chemotherapy [4]. Among the different sites of metastasis, the brain in particular presents its own unique challenges. TNBC is most prone to spread to the CNS compared to other breast cancer subtypes, and the development of TNBC brain metastases (TNBCBM) is generally associated with poor outcomes and prognosis. TNBCBM patients have a median survival time of 3.9 months, which is shorter than what is typically seen for other TNBC metastatic sites (median survival of 14.6 months) or other subtypes of breast cancer brain metastasis, such as HER2+ breast cancer (median survival of 8.0 months) [5,6].

In recent years, biomarker profiling has become an important tool for understanding TNBCBM and improving clinical care [7]. Studies have suggested microRNAs (miRs), receptor proteins, transcriptomic changes, etc., may help identify patients at a greater risk for brain metastasis and guide treatment strategies [7]. Well-known biomarkers, such as epidermal growth factor receptor (EGFR), Ki-67, and p53, have been studied in the context of TNBC, but most are linked to overall tumor aggressiveness rather than brain-specific colonization mechanisms, limiting their clinical usefulness [8]. Research progress has been slowed by two major challenges: the difficulty of obtaining CNS tissue and the frequent exclusion of patients with brain metastases from clinical trials. Despite significant research, the molecular pathways that enable TNBC cells to establish and disseminate within the brain microenvironment remain poorly understood [9].

The aim of this review is to summarize promising biomarkers that may be predictive of TNBCBM, key mechanisms involved in the transformation and neurologic dissemination of TNBC, and discuss how future research may improve early detection, prognostic accuracy, and treatment strategies for this high-risk patient population.

## 2. Materials and Methods

### 2.1. Data Collection

**Figure 1:**
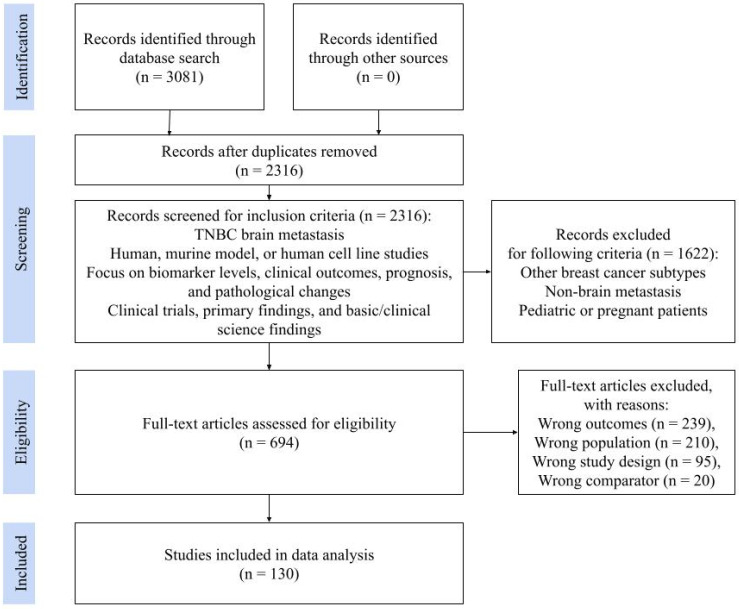
Preferred Reporting Items for Systematic Reviews and Meta-Analyses (PRISMA) diagram demonstrating article selection and screening process.

Eligible studies were identified through a literature search following Preferred Reporting Items for Systematic Reviews and Meta-Analyses (PRISMA) guidelines (Figure 1) via Covidence (Melbourne, Australia) by using key phrases, “TNBC brain metastasis,” “biomarker levels,” “prognosis,” independently and in combination with each other. Full search terms used for each database are available in Supplementary Table S2. An extensive screening process was implemented to manually review and select articles based on predefined eligibility criteria in accordance with PRISMA guidelines.

Inclusion criteria were as follows: (1) Studies including populations of human patients, murine models, human cell lines, and TNBC brain metastasis; (2) papers that analyzed biomarker levels, clinical outcomes, prognosis, and pathological changes; (3) studies including clinical trials, primary findings, and basic/clinical science experiments. Exclusion criteria were as follows: All populations of other subtypes of breast cancer, metastasis to organs other than the brain, and pediatric or pregnant patients.

Screening was carried out in two phases: title and abstract screening and full text screening. Title and abstract screening involved the evaluation of each abstract by one reviewer, while full text screening involved two independent reviewers per article followed by a mediating discussion if necessary. Articles voted for exclusion required an indication of reason for exclusion, which may include incorrect patient population, wrong outcomes, study design, etc. Conflict resolution regarding article selection encompassed opening the decision to the rest of the team to determine the inclusion or exclusion of an article to reach a consensus before proceeding to data extraction.

### 2.2. Data Extraction

Key information extracted included: presence of a control group; study population; biomarker name; category of biomarker; mechanism pathway; differences among demographics; confounding factors; presence of biomarker pre or post metastasis; percentage of TNBC population with biomarker expression; percentage of BM within the TNBC population with biomarker expression; time to metastasis (years); percentage change in biomarker expression; other numeric expression; biomarker presence/absence in other forms of breast cancer brain metastasis; method of assessment; can it be a target for treatment; and would treatment be invasive?

### 2.3. Statistical Analysis

Studies were stratified into clinical and pre-clinical, while biomarkers were categorized as clinical and radiographic features, noncoding RNA, growth factor receptors, circulating proteins, transcriptomic studies, cellular signaling and transport, immune microenvironment markers, hormone signaling, metabolism and stress signaling. Statistical analysis was done using descriptive statistics. For each biomarker category, the median and 95% CI was calculated for the percentage of TNBC populations with the biomarker and the percentage of BM within TNBC populations with the biomarker. Cis for medians were calculated differently based on sample size. For categories with *n* > 20, normal approximation methods were used, while for categories ≤ 20, exact binomial CIs based on order statistics were applied. For *n* = 3, CIs were reduced to the observed range (min-max); for *n* = 2, only the median was reported. For *n* = 1, no CI was estimable. For binary categorical data, results were reported as percentages. 

We assessed the feasibility of quantitative meta-analysis for frequently reported biomarkers, such as Ki-67 and EGFR. However, substantial heterogeneity across studies in outcome definitions (e.g., BM as first site vs. cumulative incidence vs. BM-free survival), biomarker measurement approaches (immunohistochemistry scoring systems, gene amplification or mutation status), cutoff thresholds, comparator populations, and reported effect measures (percent positivity, qualitative associations, hazard ratios, or *p*-values without extractable 2 × 2 data) precluded reliable pooling of effect estimates. Consistent with Joanna Briggs Institute guidance for systematic reviews with heterogenous evidence, formal quantitative meta-analysis and aggregate risk-of-bias scoring were not performed. Instead, study limitations and methodological considerations were assessed qualitatively and incorporated into the narrative synthesis, while risk of bias was considered qualitatively at the study level in regard to study design, comparator selection, outcome measurement, and follow-up timing.

### 2.4. Analysis of Clinical and Pre-Clinical Studies

Among clinical and pre-clinical studies, whether a biomarker was found within the TNBC population being studied was defined as “median % of TNBC population with presence of biomarker,” and the proportion of the TNBC population with the biomarker that also had brain metastases was defined as “median % of BM within the TNBC population that has the biomarker.” Due to the heterogeneity of how papers assessed the biomarkers in TNBCBM, we encapsulated the difference in the amount, presence, or clinical/radiographic manifestation of the biomarker as “significant difference in biomarker,” defined as the percentage of papers that observed a significantly higher level or association of the biomarker within the TNBC population with BM as compared to the study’s control population. We also quantified therapeutic potential as “is it/can it be a target for treatment?” as a percentage of biomarkers that can be targeted for therapy (% Yes) and percentage of biomarkers that cannot (% No) based on whether there is a therapeutic—at least in clinical development if not available on the market—which targets the biomarker. Additionally, the percentage of biomarkers within a category that were present before or after metastasis, or both, was defined as “% primary; % metastasis; or % both”. For each percentage value, the number of biomarkers that were used to calculate the percentage was given as *n* = #.

## 3. Results

### 3.1. Study Characteristics

From an initial search yielding 2316 unique articles, 694 full-text articles were assessed for eligibility after exclusion of records that did not pertain to biomarkers of TNBCBM, and a total of 130 studies were included after screening for study design and demographics (Figure 1). The included publications consisted of 60 clinical and 70 pre-clinical investigations (Table 1). Clinical investigations were characterized by analyses of human patients or human-derived tissue, whereas pre-clinical investigations involved animal models or in vitro cell-line systems. This distinction provided a clearer framework for standardizing reported outcomes and facilitated more meaningful comparisons across heterogeneous study designs.

The studies that contributed to the data vary in sample size, methodologies, and geographic location, which may account for variability in prevalence estimates across studies. Additionally, biomarkers were classified based on their role in tumor progression, metastatic potential, or therapeutic response. The designed scheme provided an important comparison between the general TNBC population and those with brain metastases, helping to identify potential biomarkers that are more strongly associated with metastasis to the brain.

From the 130 studies that met our search criteria, clinical and pre-clinical studies investigated a total of 93 and 79 biomarkers, respectively, with some studies assessing multiple biomarkers. As a comparison for TNBCBM, the majority of studies included a comparison group such as primary TNBC tumors (87% of studies), non-TNBC breast cancer brain metastases (7%), and TNBC metastasis to regions other than the brain (6%). Assessment of these studies showed that 73.6% of the clinical and 94.9% of the preclinical studies showed a comparison to one or more of these groups (Table 1). Clinical and preclinical studies were then analyzed with respect to clinical and radiographic features, noncoding RNA, growth factor receptors, circulating proteins, transcriptomic studies, cellular signaling and transport, immune microenvironment markers, hormone signaling, or metabolism and stress signaling, with the top five biomarkers based on prevalence highlighted (Supplemental Table S1).

### 3.2. Key Findings

#### 3.2.1. Clinical and Radiographic Features

**Figure 2:**
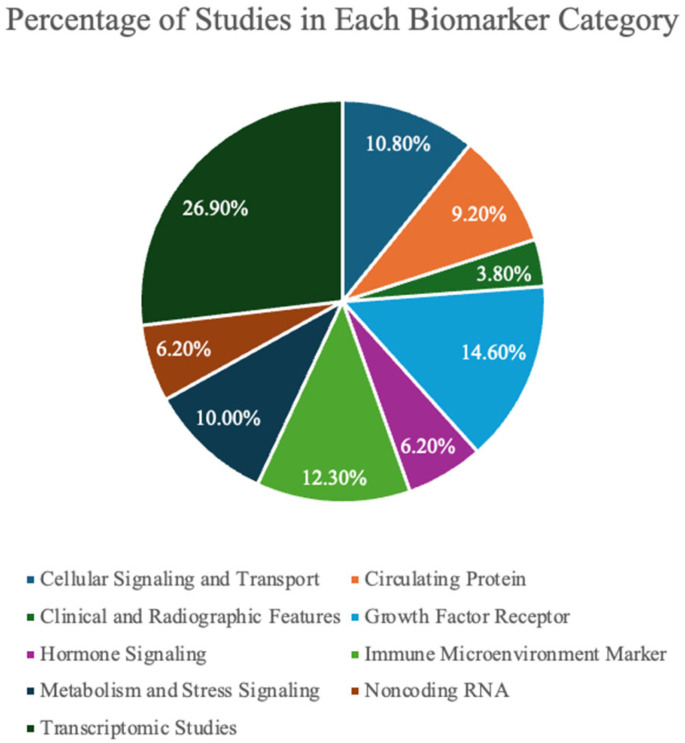
Percentage of publications by biomarker category. Studies were categorized based on the biomarker being studied, and percentages were determined by proportion in each category to the total number of studies (*n* = 130).

**Figure 3:**
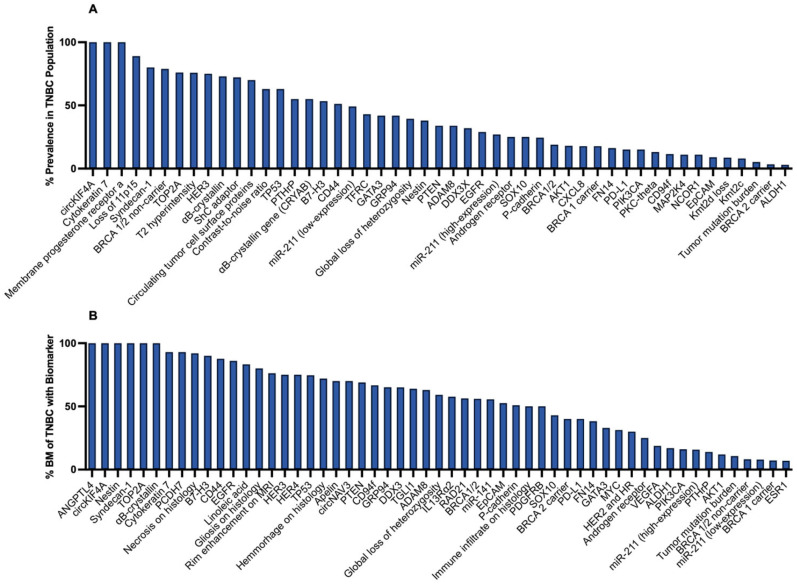
Quantitatively reported biomarker expression in (**A**) TNBC and (**B**) TNBC brain metastases. This figure displays the biomarkers with the highest quantitatively measured expression across the included studies. Only biomarkers for which extractable numerical data (e.g., percentage expression, positivity rate, or incidence within the sampled population) were available were plotted. For biomarkers evaluated in more than one study, the mean percentage reported across those studies was calculated. (**A**) Bars represent the percentage of TNBC cases expressing each biomarker, based on the study populations that assessed that specific marker. (**B**) Bars represent the percentage of TNBC brain metastasis samples expressing the biomarker, reflecting their proportional expression within metastatic tissue. By comparing expression levels in (**A**) primary TNBC tumors and in (**B**) TNBC brain metastases, this figure highlights biomarkers that may be enriched or depleted during metastatic progression. Biomarkers shown include both clinically validated markers (e.g., EGFR and PD-L1) and biomarkers currently supported primarily by preclinical evidence (e.g., circulating proteins such as ANGPTL4). Biomarkers mentioned elsewhere in the manuscript but lacking standardized quantitative data were excluded from this figure, even if they were frequently discussed qualitatively in the literature.

Several clinical and radiographic features demonstrated prognostic value in TNBCBM (Supplemental Table S1), with 3.8% of all studies investigating this biomarker category (Figure 2). Lymph node metastasis was a strong predictor of TNBCBM [10,11,12]. Similarly, the Ki-67 proliferation index showed increasing risk with higher expression levels [11,12]. Radiographically, T2 hyperintensity and lesion rim enhancement on magnetic resonance imaging (MRI) correlated with TNBCBM, reflecting vasogenic edema and blood–brain barrier disruption [14,71] (Figure 3B). Rim enhancement was observed in 67.7% of TNBCBM lesions compared to 29% of receptor-positive brain metastases [140] (Figure 3B). Additionally, the contrast-to-noise ratio (CNR) provided a quantitative MRI parameter to correlate metastatic lesion composition, with higher CNR representing clearer demarcation of tumor from surrounding tissue [71]. A total of 63% of primary tumors were found to have high CNR (Figure 3A) suggesting that CNR may serve as a noninvasive imaging biomarker for identifying or characterizing metastatic potential and tumor biology in TNBC.

#### 3.2.2. Noncoding RNAs

Multiple noncoding RNAs demonstrated diagnostic and mechanistic relevance in TNBCBM, and RNAs were binned based on the number of patients who expressed the marker (Supplemental Table S1). Out of all the studies, 6.2% of them investigated this biomarker category (Figure 2). Among the noncoding RNAs identified, circular RNA Kinesin Family Member 4A (*circKIF4A*) was significantly upregulated in 100% of patients analyzed. Studies by Wu et al. proposed that *circKIF4A* initiates a cascade that downregulates *STAT3* via suppression of *miR-637*, creating an axis that is important for metastatic coordination [78] (Figure 3A,B). Another miR identified with high prevalence in TNBCBM patients was *miR-211. miR-211* showed strong predictive value for brain metastasis by receiver operating characteristic analysis (Figure 3A,B), with recent investigations demonstrating that *miR-211* exerts a multifaceted effect on TNBC cells [74]. Mechanistically, *miR-211* has been shown to enhance the intrinsic migratory capabilities of tumor cells by modulating key cellular pathways that govern cell adhesion, invasion, and survival in the hostile microenvironment of the brain [74]. Specifically, high *miR-211* expression increased the ability of tumor cells to adhere to endothelial cells lining the blood–brain barrier (BBB) as well as facilitating transendothelial migration to these tumor cells [74]. Conversely, transcriptomic profiling revealed significant downregulation of *miR-802-5p* and *miR-194-5p* in mouse studies [75,77]. Both miRs were predicted to target *MEF2C*, a transcription factor implicated in various cellular processes such as development, differentiation, cell survival, and stress responses. Finally, loss of *miR-623* expression was implicated in TNBCBM. *miR-623* has been shown to target metalloproteinase-1, a driver of extravasation, and was downregulated in TNBCBM compared with primary breast tumors. Restoring *miR-623* reduced transendothelial migration in TNBC models, suggesting a mechanism for *miR-623* involvement in metastasis generally [73].

#### 3.2.3. Growth Factor Receptors

Several growth factor receptors and their associated pathways were implicated in TNBCBM (Supplemental Table S1), with 14.6% of all studies assessing this biomarker category (Figure 2) [14,15,16,17,18,19,20,21,80,81,82,83,84,85,86,87,88,89,90]. Among the myriads of molecular drivers contributing to TNBC metastatic progression, receptor tyrosine kinases (RTKs) centering around EGFR signaling have garnered significant attention. Human epidermal growth factor Receptor 3 (HER3), c-mesenchymal–epithelial transition factor (c-MET), the adaptor protein Src homology and collagen D (shcD), and loss of the tumor suppressor phosphatase and tensin homolog (*PTEN*) were among the most common proteins cited relative to TNBCBM. Elevated EGFR expression in these lesions was closely associated with enhanced receptor dimerization and subsequent autophosphorylation—events that trigger downstream signaling cascades critical for cell survival, proliferation, and invasion. EGFR plays a central role in activating two major downstream pathways, namely the Phosphatidylinositol 3-kinase (PI3K)/Protein Kinase B (Akt) and the mitogen-activated protein kinase (MAPK) pathways [141]. Upon ligand binding or via ligand-independent mechanisms promoted by adaptor proteins, EGFR undergoes autophosphorylation on several tyrosine residues. This phosphorylation event provides docking sites for multiple adaptor proteins and initiates a cascade of intracellular events, influencing the transcriptional regulation and metabolic reprogramming of cancer cells [142]. HER3 commonly forms heterodimers with other HER family members such as EGFR or HER2. These heterodimeric complexes potentiate the signaling output, even in the presence of low ligand concentrations. Notably, HER3 activation has been implicated in resistance to EGFR-targeted therapies, as compensatory signaling via HER3 can maintain PI3K/AKT activation in the presence of EGFR inhibition. In TNBC, where EGFR is overexpressed in 55% of patients, the interplay between EGFR and HER3 creates a formidable signaling unit that drives metastasis and therapeutic resistance [143]. In TNBCBM, EGFR expression was markedly elevated in metastatic lesions compared with primary TNBC tissue, with metastatic lesions showing membranous and cytoplasmic EGFR expression [17,18,84] (Figure 3A,B). EGFR inhibition by afatinib prolonged survival and effectively treated the lesions, whereas its activation increased propensity for brain metastasis [84]. Similarly, HER3 positivity was seen in 75% of TNBCBM lesions, with metastatic lesions showing a significant upregulation compared to the primary tumors [14] (Figure 3A,B).

c-MET, the receptor for hepatocyte growth factor, is another major player in oncogenic signal transduction frequently implicated in TNBCBM [144]. Similar to EGFR, activation of c-MET can independently trigger the PI3K/AKT and MAPK pathways [145]. The crosstalk between c-MET and EGFR has significant implications for tumor biology. Both receptors can converge on the same intracellular signaling routes, thereby providing cancer cells with multiple avenues to achieve survival, migration, and proliferation [82,87]. This redundancy in signaling not only enhances oncogenic potential, but also poses a considerable challenge for targeted therapy, as inhibition of one receptor often results in compensatory activation of the other [146,147].

Another novel modulator for EGFR signaling is ShcD, a member of the Src homology and collagen (Shc) family of adaptor proteins. Unlike its more extensively studied homologs, ShcD exhibits unique properties that allow it to enhance EGFR phosphorylation even in the absence of ligand stimulation [148]. This phenomenon is particularly relevant in TNBC, where ShcD is frequently upregulated and correlates with aggressive tumor behavior and metastasis [90] (Figure 3A).

Finally, several studies have highlighted the loss of *PTEN* in TNBCBM. *PTEN* is a well-known tumor suppressor that negatively regulates the PI3K/AKT pathway by dephosphorylating phosphatidylinositol 3,4,5-trisphosphate into phosphatidylinositol 4,5-bisphosphate. In normal cells, *PTEN* activity is critical for maintaining cellular homeostasis by keeping PI3K/AKT signaling in check. Loss or mutation of *PTEN* results in constitutive activation of the PI3K/AKT pathway, promoting cell survival, proliferation, and migration. The absence of *PTEN* activity amplifies the oncogenic signals initiated by RTKs such as EGFR and c-MET, leading to a hyperactivated PI3K/AKT pathway [149]. *PTEN* mutations occurred more frequently in brain metastases (69%) than in primary breast cancer lesions (34%) (Figure 3B), with *PTEN* loss showing a stronger correlation with the brain than with extracranial metastatic sites [18]. 

#### 3.2.4. Circulating Proteins

Circulating tumor cells, vesicles and/or DNA have demonstrated diagnostic and mechanistic relevance in TNBCBM (Supplemental Table S1). Of the included studies, 9.2% investigated this biomarker category (Figure 2). Circulating tumor cells (CTCs) detected after enrichment by both the Epithelial Cell Adhesion Molecule (EpCAM)-dependent and independent method were commonly observed in TNBC patients. Interrogation of these cells revealed that 60% of TNBC patients exhibited CTCs positive for both EGFR and keratins and 40% positive for keratins only, and the presence of CTCs significantly associated with decreased survival after brain metastasis diagnosis [23] (Figure 3A).

Several studies have highlighted the usage of secreted proteins for TNBCBM diagnosis and extravasation into the CNS, with angiopoietin-like 4 (ANGPTL4) proposed to be one of the top biomarkers found. Studies by Simeon et al. showed that 89% of mice with TNBC that had high expression of ANGPTL4 developed brain metastasis (BM) (Figure 3B) [94]. ANGPTL4 is a secreted glycoprotein commonly observed in TNBC, and the overexpression of ANGPTL4 in cell lines led to increased numbers and size of mammospheres. These mammospheres expressed lower levels of cluster of differentiation 24 (CD24) and contained more lipid droplets [94]. Interestingly, TNBC patients with ANGPTL4 enhancement inhibited cell migration [150]. Thus, this protein should be further explored to investigate its impact on TNBC metastasis.

Another secreted protein observed in TNBCBM is Serpin Family B Member 1 (SERPINB1), a member of the serpin superfamily of proteins. SERPINB1 is secreted and involved in regulating protease activity throughout the body. Studies show that SERPINB1 is elevated only in brain metastases as compared to primary tumors or other distant metastatic lesions, with upregulation associated with a lower progression-free survival [96]. Finally, among the top circulating biomarkers published, Hyaluronidase 1 (HYAL1) expression was higher in primary tumors of BM patients but significantly lower in BM compared with both primary and other metastatic sites [25]. HYAL1 is an enzyme involved in hyaluronan metabolism. Data suggests that HYAL1 produces fragments of hyaluronan that help cancer cells stick to and break through the BBB. In TNBC, low-molecular-weight forms of hyaluronan appear to coat breast cancer cells, in turn promoting tumor cell adhesion, disruption, and migration through the brain endothelium [112].

In addition, loss of several biomarkers correlated with increased TNBCBM. In TNBC, loss of the protein AT-rich interaction domain 1A (*ARID1A*), a core component of the Switch/Sucrose Non-Fermentable (SWI/SNF) chromatin remodeling complex, may influence tumor biology and the systemic signals reflected in patient serum. Loss or mutation of the *ARID1A* gene can alter transcriptional programs governing differentiation, DNA damage response, and epigenetic regulation, potentially modulating the release of cytokines, growth factors, and extracellular vesicles that shape the serum proteome and circulating tumor DNA/RNA signatures [151]. In metastatic TNBC, *ARID1A* mutations occur in 7.1% of patients and has been associated with changes in chromatin accessibility and gene expression that could affect immune signaling, metabolism, and stromal interactions, all of which may impact the serum biomarkers that are used for prognosis or therapy monitoring [152]. Over 80% of TNBCBM patients showed loss of *ARID1A* expression [94]. 

Finally, several studies utilized exosomes to identify biomarkers for metastatic seeding to the brain. Exosomes are small, membrane-bound extracellular vesicles, typically about 30–150 nanometers in diameter, released by nearly all cell types. Exosomes carry a cargo of proteins, lipids, and nuclear material including mRNA and miR, that reflect the cell of origin and can influence recipient cells by transferring this cargo. Ongoing research has shown that annexin A2 (AnxA2) was elevated in cancer exosomes versus non/pre-malignant exosomes, and high exo-AnxA2 correlated with increased metastatic disease compared to non-metastatic breast cancer [24] and its presence induced angiogenesis. Conversely, genetic knockdown of AnxA2 reduced brain metastasis by approximately 30% [24]. 

#### 3.2.5. Transcriptomic Studies

Genomic and transcriptomic analyses revealed several recurrent molecular alterations associated with TNBCBM (Supplemental Table S1), with 26.9% of the included studies examining this biomarker category (Figure 2). *TP53* mutations were prevalent, seen in an average of 69% of primary TNBC and about 80% of TNBCBM, with mutations usually matching between primary and BM lesions (Figure 3A,B). Among breast cancer subtypes, TNBC accounted for the largest share of tumor protein p53 (*TP53*) mutations in BM [27,28,30,31,35,45]. Correlation of *TP53* mutations with level of expression, either increased or decreased, was not always explicitly discussed due to the variety of genomic alterations found, with certain mutations corresponding to a loss of p53 function [27] while others stabilized and increased p53 expression [35]. Genomic studies also revealed that an allelic imbalance at chromosome 11p was significantly associated with the development of breast cancer. Specifically, loss of 11p15 was commonly observed across all breast cancer subtypes [34] and was commonly observed in patients with triple-negative status. A total of 89% of TNBC cases showed an allelic imbalance at 11p15 (Figure 3A), which was observed in both primary TNBC and BM formation. Finally, BM was observed in 40% of Breast Cancer gene 2 (*BRCA2*) germline pathogenic variant carriers. While not significant, *BRCA2* carriers had an odds ratio of 1.75 for TNBC patients with BM, with BM occurring as the first metastatic in 26.7% of patients within the study cohort [32] (Figure 3B). Transcriptomic studies indicated positivity of several other BM transcripts including cytokeratin 7 and the transcription factors GATA-binding protein 3 (*GATA3*) and SRY-box transcription factor 10 (*SOX-10*). In TNBCBM samples, 96% cytokeratin 7 positivity, 77% *GATA3* positivity, and 15% *SOX-10* positivity were seen, with at least one of cytokeratin 7, *GATA3*, or *SOX-10* transcript present in all TNBCBM samples [44] (Figure 3B). 

#### 3.2.6. Cellular Signaling and Transport

Several cellular signaling and transport biomarkers were identified as contributors to TNBCBM formation and progression (Supplemental Table S1), and many proteins related to adhesion were among the top five cited in TNBCBM; 10.8% of the included studies investigated this biomarker category (Figure 2). Pharmacologic inhibition of integrin signaling resulted in reduced cellular growth, migration, and invasion, with the corresponding suppression of downstream signaling components [111,113,114]. Syndecan-1, a transmembrane protein involved in cell adhesion and signaling through its heparan sulfate chains, was expressed in all metastatic lesions, ranging from a moderate to strong membrane pattern of expression. This is consistent with previous studies that have shown that the downregulation of syndecan-1 expression significantly reduced brain colonization by inhibiting migration across the blood–brain barrier and adhesion to perivascular regions within the brain [53,116] (Figure 3B). Finally, A Disintegrin and Metalloproteinase domain 8 (ADAM8) was localized to the cytoplasm and plasma membrane of cancer cells, but it was not present in the adjacent normal mammary tissue. In primary TNBC, its expression was detected in 34% of tumor cells, particularly at the microinvasive front (Figure 3A). Knockdown or anti-ADAM8 treatment resulted in a decrease in average tumor weight and a ~50% decrease in primary tumor burden [117].

Outside of genes related to adhesion, specific receptors were commonly identified in TNBCBM. Increased gene expression of the transferrin receptor was observed across breast cancer subtypes but was highest in primary TNBC, with comparable expression observed between primary and TNBCBM [118]. Finally, high parathyroid hormone-related protein (PTHrP) expression was found in ~55% of TNBC cases. High expression of PTHrP in the primary tumor correlated with increased brain metastasis and acted as an independent prognostic factor for CNS-progression-free survival (CNS-PFS) [50]. Although this marker was not an independent prognostic factor for overall survival (OS), patients with high PTHrP expression showed a significant reduction in the 5-year CNS-PFS (81% versus 97% for low PTHrP groups) and brain metastasis (84% versus 100% for low PTHrP groups) [50].

#### 3.2.7. Immune Microenvironment Markers

Multiple immune-associated pathways were found to influence TNBCBM formation and progression (Supplemental Table S1); 12.3% of the included studies investigated this biomarker category (Figure 2). Immune checkpoint inhibitors, particularly involving the Programmed Cell Death Protein-1 (PD-1)/Programmed Death-Ligand 1 (PD-L1) pathways, have gained traction in the clinical management of multiple cancers due to their ability to activate the immune system to eliminate cancerous cells [153], accounting for 2.3% of the included publications on BM in TNBC (Supplemental Table S1). Since 2020, the Food and Drug Administration (FDA) approved usage of PD-1/PD-L1 therapy for TNBC has shown that up to 20% of patients are PD-L1 positive. Of these patients, across two studies, an average of 25% of BM expressed PD-L1 [49,60] (Figure 3B). Similarly, another checkpoint inhibitor target B7 homolog 3 protein (B7-H3), has recently gained traction as a novel modulator of the tumor microenvironment [154]. B7-H3 expression was significantly higher in TNBC versus ER+ breast cancer, and in TNBC, it was associated with high Ki-67 expression (Figure 3A). In TNBCBM, 90% of samples were found to be positive for this biomarker, suggesting it may serve as a novel therapeutic target [58] (Figure 3B).

Multiple cell populations within the tumor microenvironment have been implicated in promoting BM. Protein kinase C θ (PKC-θ), a serine/threonine kinase, was highlighted in 1% of papers, suggesting a role in BM. PKC-θ is purported to be highly expressed in cancer stem cell-enriched TNBC cells, and studies have also shown that it is expressed in mesenchymal circulating tumor cells as well as in dysfunctional cluster of differentiation 8 positive (CD8^+^) T cells within the brain metastatic microenvironment [128] (Figure 3A). In addition, cytokines were highlighted in several papers. The proinflammatory cytokine interleukin-1 beta (IL-1β) expression correlated with metastatic lesion size, with higher expression also correlating with disruption of the BBB and TNBC transmigration into the brain [120,122]. Astrocyte IL-1β expression was elevated ~10× when exposed to TNBC cells and ~30× when cultured with factors secreted by BM cells. Moreover, neutralization of IL-1β decreased breast cancer proliferation in vitro in both human and mouse models [120,122]. Platelet Endothelial Cell Adhesion Molecule (PECAM) was significantly upregulated (≥1.5-fold) in breast cancer cell lines, with a 2.5-fold increase compared to non-metastatic cells [124].

#### 3.2.8. Hormone Signaling

Alterations in hormone receptor expression and signaling were frequently observed in TNBCBM, accounting for 6.2% of publications that met our inclusion criteria (Figure 2). Given that TNBC is ER, PR, and HER2 negative, this suggests a new emphasis on potential subtype plasticity and responsiveness to hormone signaling (Supplemental Table S1). Approximately 13% of TNBC lesions that had BM showed conversion to progesterone receptor (PR) positivity. In addition, while TNBC tumors lack nuclear expression of PR, they have been shown to express membrane progesterone receptor α (mPRα) [133]. mPRα was expressed in TNBC lesions, and its levels were negatively correlated with metastatic potential (Figure 3A) [61,133]. Moreover, significant plasticity was observed for other hormone receptors; 13–22% of TNBC lesions that metastasized to the brain displayed subtype switching to ER positivity in monocentric studies [61,63] (Figure 3B). In mouse studies, 100% of mice treated with estrogen displayed TNBCBM and showed decreased survival compared to 60% of the control group showing TNBCBM. In multiple studies, treatment with estrogen increased rates of BM by 3.6 to 21-fold in mice models using a variety of TNBC cell lines [129,130,132]. Finally, both Androgen receptor and HER2 was upregulated in TNBCBM. Androgen receptor was expressed in 25% of TNBCBM lesions [62] (Figure 3B) while gain of HER2 receptor positivity occurred in approximately 5% of TNBCBM lesions [61,63]. The change in receptor status for many of these receptors is intriguing and offers a potential method to treat TNBCBM given the number of drugs available. 

#### 3.2.9. Metabolism and Stress Signaling

Multiple metabolic and stress signaling pathways were implicated in the progression and poor prognosis of TNBCBM, and this accounted for 10.8% of the publications that met our inclusion criteria (Figure 2). In particular, regulation of proteostasis pathways accounted for many of the proteins that were commonly cited. High expression of αB-crystallin and Glucose-Regulated Protein 94 (GRP94) were commonly observed and predicted to act as key chaperone-based stabilizers of proteome integrity under stress. Dead-box helicase 3 (DDX3)—an RNA helicase that is frequently cited—linked RNA metabolism and stress-responsive translation to the maintenance of oncogenic programs. Several studies have shown that patients with αB-crystallin positive primary tumors showed poor OS [65,68,136] (Figure 3B). Increased expression of αB-crystallin was observed in basal-like TNBC versus non-basal TNBC and was maintained between the primary tumor and BM (37–73% versus 47–67%), serving as an independent prognostic factor for breast cancer-specific survival (a measure that excludes deaths from causes other than breast cancer) (Figure 3A). Additionally, GRP94 overexpression was found in 41.9% of TNBC tumors (Figure 3A), with higher expression levels in TNBC patients who developed BM compared to non-metastatic groups (65.1% vs. 42.2%; Figure 3B) and significantly shorter BM-free survival [64,137].

These changes in proteostasis are complemented by changes in the hypoxic response, whereby both networks appeared to converge to support tumor survival and progression. Differential gene expression analyses identified the upregulation of pathways involved in hypoxic signaling, angiogenesis, and glycolysis, with higher expression of Hypoxia Inducible Factor 1-α (HIF1α) targets found to be associated with reduced OS [67]. HIF1α-driven transcriptional reprogramming promotes glycolysis, angiogenesis, and cell survival in hypoxic niches. Nuclear receptors added another regulatory layer, modulating transcriptional programs that intersected with HIF1α targets and stress-response pathways, thereby shaping metabolic and apoptotic contexts favorable to TNBC cell survival, invasion, and therapy resistance. Together, these factors formed an integrated proteostasis-hypoxia-transcription axis in TNBC, where DDX3-driven translation of stress-responsive mRNAs and nuclear receptor-mediated transcription could synergize with GRP94 and αB-crystallin to sustain malignant phenotypes under adverse microenvironmental and chemotherapeutic pressures. Supporting this supposition, upregulated cytoplasmic DDX3 expression was found in 28% of metastatic breast cancers, most frequently in TNBC (and in 65% of TNBCBM lesions) and was associated with worse OS [88] (Figure 3B). Finally, TNBC cell lines had a 4× higher uptake of nicotinamide riboside compared to other cell lines, with nicotinamide riboside supplementation significantly increasing brain metastasis formation in TNBC mouse models [134]. Targeting these interconnections offers a rationale for combinatorial strategies to counter TNBC aggressiveness.

### 3.3. Pre-Clinical Studies Analysis

To further compare all the biomarkers across all categories, we first analyzed the biomarkers identified in pre-clinical publications (Table 2). We note that there is a discrepancy in sample sizes across different variables due to the variability in how each study reported its findings. Key findings still emerged from the analysis, despite the lack of consistent sample sizes across all included studies.

Initially, the median percentages of biomarker positivity in primary TNBC was binned according to the following categories: cellular signaling and transport (38.5% of TNBC samples), circulating protein (N/A), clinical and radiographic features (69.4%), growth factor receptor (72.2%), hormone signaling (100.0%), immune microenvironment marker (13.1%), metabolism and stress signaling (73.0%), noncoding RNA (49.2% [95% confidence interval {CI} 27.0–100.0%]), and transcriptomic studies (9.0% [95% CI 8.7–24.5%]) (Table 2). Similarly, the median percentage of TNBC samples expressing the biomarker which displayed BM were reported based on the categories of cellular signaling and transport (63.0%), circulating protein (100.0%), clinical and radiographic features (N/A), growth factor receptor (53.9%), hormone signaling (83.3%), immune microenvironment marker (70.0%), metabolism and stress signaling (N/A), noncoding RNA (55.6% [95% CI 7.9–100.0%]), and transcriptomic studies (64.0% [95% CI 50.9–87.7%]). For whether the biomarker was present in the primary tumor, metastatic lesion, or both, primary tumor-only expression was commonly observed for clinical and radiographic features (100%), noncoding RNA (100%), hormone signaling (100%), transcriptomic studies (89.5%), immune microenvironment markers (88.9%), cellular signaling and transport (71.4%), growth factor receptors (72.7%), circulating proteins (62.5%), and metabolism and stress signaling (66.7%); metastatic lesion-only expression was less common across categories, seen in circulating proteins (37.5%), metabolism and stress signaling (33.3%), growth factor receptors (18.2%), cellular signaling and transport (14.3%), transcriptomic studies (10.5%), and immune microenvironment markers (11.1%); biomarkers present in both primary tumors and metastatic lesions were less common, occurring in only growth factor receptors (9.1%) and cellular signaling and transport (14.3%) (Table 2).

We next characterized individual biomarkers with regard to their therapeutic potential. This was quantified based on the proportion of biomarkers identified as targetable by existing or investigational therapies. Cellular signaling and transport (87.5%), hormone signaling (75.0%), and growth factor receptors (66.7%) were the most targetable categories, followed by immune microenvironment markers (60.0%), transcriptomic studies (47.8%), metabolism and stress signaling (45.5%), circulating proteins (37.5%), noncoding RNA (18.8%) and clinical and radiographic features (0%).

### 3.4. Clinical Studies Analysis

We ran similar analyses for all biomarkers included in clinical studies (Table 3). The median percentage of TNBC populations with the biomarker were categorized as follows: cellular signaling and transport (67.5%), circulating protein (70.0%), clinical and radiographic features (N/A), growth factor receptor (33.0% [95% CI 16.2–75.0%]), hormone signaling (30.0%), immune microenvironment marker (34.2%), metabolism and stress signaling (48.5% [95% CI 32.0–73.0%]), and transcriptomic studies (25.0% [95% CI 15.5–38.0%]). The median percentage of BM within the TNBC population with the biomarker segregated based on these categories is as follows: cellular signaling and transport (57.0%), circulating protein (no studies), clinical and radiographic features (76.2%), growth factor receptor (75.0% [95% CI 71.0–86.0%]), hormone signaling (65.0%), immune microenvironment marker (76.0% [95% CI 50.0–90.0%]), metabolism and stress signaling (65.1% [95% CI 65.0–100%]), and transcriptomic studies (37.5% [95% CI 16.0–56.3%]). For whether the biomarker was present in the primary tumor, metastatic lesion, or both, primary tumor-only expression was most commonly observed for transcriptomic studies (63.8%), cellular signaling and transport (66.7%), circulating proteins (50.0%), hormone signaling (50.0%), clinical and radiographic features (75.0%), and metabolism and stress signaling (37.5%); metastatic lesion-only expression was observed most frequently for growth factor receptors (85.7%), metabolism and stress signaling (62.5%), immune microenvironment markers (45.5%), circulating proteins (50.0%), hormone signaling (50.0%), and cellular signaling and transport (33.3%); biomarkers present in both primary tumors and metastatic lesions were less common, occurring in transcriptomic studies (21.3%) and immune microenvironment markers (18.2%) (Table 3). We next evaluated the therapeutic potential of these biomarkers using the criteria above and found hormone signaling (83.3%) to have the highest potential for targeting. This was followed by circulating proteins (75.0%), growth factor receptors (66.7%), and then cellular signaling and transport (66.7%). Transcriptomic studies (58.3%), metabolism and stress signaling (57.1%), and immune microenvironment markers (54.5%) showed moderate levels of targetability, whereas clinical and radiographic features demonstrated 0% targetability.

**Figure 4:**
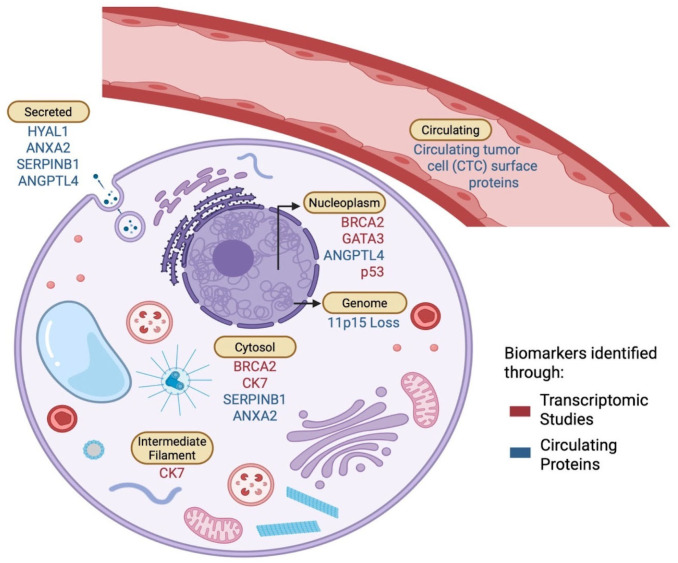
Localization of candidate biomarkers identified in triple-negative breast cancer brain metastases (TNBCBM). Biomarkers were mapped to cellular and systemic compartments based on protein and transcriptomic localization data (Human Protein Atlas). Circulating proteins (blue) are primarily secreted, while transcriptomic biomarkers (red) localize to nuclear and cytoplasmic compartments. The chromosomal loss at 11p15 is represented at the genomic/chromatin level. This schematic summarizes biomarker distribution and potential functional pathways linking the primary tumor, circulation, and metastatic niche. Created in BioRender. Wadehra, M. (2026) https://BioRender.com/kwskmp7 (accessed on 12 February 2026).

Building on the most frequently observed biomarker categories, we next prioritized biomarkers with direct therapeutic relevance. Actionable biomarkers, corresponding targeted therapies, and relevant clinical trials are summarized in Supplementary Table S3. From our analyses, transcriptomic studies and circulating proteins were the most represented across all included studies and sensitive biomarkers showing nonsignificant yet increased expression in TNBCBM compared to the primary tumor. A conceptual schematic (Figure 4) summarizes the subcellular and systemic localization of the top five biomarkers from these two categories and illustrates their potential interactions between the primary tumor, circulation, and brain metastases. Circulating biomarkers, primarily secreted proteins, are represented in blue (Figure 4). These proteins are found primarily in the bloodstream and are thought to play significant roles in promoting the metastatic spread of TNBC cells. The secretion of these biomarkers into circulation may influence communication between the primary tumor and distant metastatic sites, including the brain. Their presence in circulation makes them potential candidates for liquid biopsy approaches to monitor disease progression or response to treatment. On the other hand, transcriptomic biomarkers are localized to the nuclear and cytoplasmic compartments of cells and are represented in red (Figure 4). Their expression reflects gene activity at the transcriptional level, which may correlate with the activation of signaling pathways that are crucial for the survival and growth of metastatic cells. Nuclear localization is particularly important for understanding the regulation of gene expression related to tumor progression, while cytoplasmic localization may suggest involvement in processes such as cell motility, survival, or response to microenvironmental cues. A key genomic alteration, chromosomal loss at 11p15, is also highlighted at the genomic/chromatin level. This loss may contribute to the pathogenesis of TNBCBM by disrupting the regulation of genes involved in cellular growth and survival. This emphasizes the complex interplay between genetic alterations and their impact on protein expression and localization, which can drive the metastatic process.

## 4. Discussion

Across our analyses, both clinical and preclinical studies reveal that circulating protein biomarkers are the most consistently expressed and enriched in TNBC patients who develop brain metastasis. This shows that despite differences in study design and patient cohorts, protein biomarkers prevail as holding the strongest predictive potential. Importantly, many of the most frequently reported circulating protein biomarkers (e.g., Annexin A2, ANGPTL4) in our review have been studied in a preclinical or exploratory context, where they provide valuable mechanistic insight into BBB disruption, tumor-microenvironment interactions, and metastatic colonization [24,94,150]. This may be due to multiple non-exclusive factors. Firstly, TNBC’s aggressive phenotype and lack of hormone receptors may lead to overreliance on alternative tumorigenesis pathways such as growth factor signaling, cell adhesion, and extracellular matrix proteins [155,156,157,158]. This is most likely reflected in the biomarkers associated with the tumor’s intrinsic biology as well as its interactions with its microenvironment, which are key in the metastatic colonization of the brain [71]. In contrast, several biomarkers such as PD-L1 and EGFR have been directly evaluated in human TNBC brain metastatic tissue, which may provide greater translational relevance. For example, PD-L1 expression has been reported in close to 25% of TNBC brain metastatic specimens [49,60], and EGFR alterations have been observed in both primary tumor and matched brain metastases [17,18,84]. Thus, circulating protein biomarkers hold significant importance: a significant association with tumor aggression and future metastasis can aid in risk stratification for CNS surveillance and prophylactic therapies.

Although pooled effect estimates—such as odds ratios—were considered for commonly studied biomarkers (e.g., Ki-67 and EGFR), heterogeneity in study design, biomarker definitions, and outcome reporting limited the interpretability of any formal meta-analytic summary. In silico analysis was used to map biomarker distribution across the tumor’s primary site, the circulation, and the metastatic brain niche, while also proposing potential pathways by which these biomarkers may influence disease progression (Figure 3). These interactions between compartments are crucial for understanding the dynamic nature of TNBCBM, which involves the interplay between circulating factors, tissue-specific signaling, and genomic changes that together facilitate the growth and colonization of brain metastases. This framework also highlights the distinction between biomarkers that primarily elucidate metastatic biology versus those with emerging clinical applicability (Supplementary Table S3). The identification and study of these biomarkers could open new avenues for therapeutic strategies aimed at preventing or treating brain metastasis in TNBC patients.

Due to the array of biomarkers that exist in TNBC, treatment for this disease is incredibly heterogeneous. Thus, many different markers can be targeted for therapy, highlighting the importance of understanding the existing signaling pathways. If we can clearly understand these markers, we can personalize and individualize treatments that are specific to a patient’s needs, likely increasing their response rates to treatment. We show that circulating protein biomarkers are not only common among TNBC patients and have a relatively high rate of correlation with further BM development, but that they can also be incorporated into clinical practice rather easily through methods such as proteomics. Beyond tissue-based profiling, liquid biopsy approaches such as CTCs and tumor-derived exosomes are currently being studied in clinical trials and offer a minimally invasive strategy for both the early detection and longitudinal monitoring of BM, particularly in circumstances where repeated intracranial sampling is not feasible [159,160,161]. Additionally, given that we found the majority of circulating protein biomarkers to be present prior to metastasis, our results can better inform providers of the predicted risk of brain metastasis in their patients, given the presence or absence of certain biomarkers, allowing for improved shared decision-making and more effective clinical interventions. To further support our findings, future prospective longitudinal studies should validate these biomarkers as well as their associated risk determinant by integrating machine learning with biomarker and disease progression data/imaging to create risk prediction models. Furthermore, exploration into the applicability of circulating protein biomarkers in informing the selection of treatment targets can allow them to hold a dual predictive and therapeutic role. This will then allow for the parallel development of preventive interventions for those identified as high-risk for BM earlier in their disease trajectory. Notably, several actionable biomarkers summarized in Supplemental Table S3 (e.g., EGFR, PD-L1, HER3) are linked to targeted or immune-based interventions with ongoing or completed clinical trials that include patients with CNS involvement, highlighting their potential clinical applicability in the near future. For example, there are no current medications targeting ANGPTL4 on the market, however there are various in clinical development such as MAR001 and REGN1001 [162,163,164]. Similarly, preclinical inhibitors of Annexin A2, such as hexapeptides and small molecule inhibitors, have been developed to target neoplastic growth in animal models but are not yet approved for humans [165,166]. In parallel, BBB-penetrant strategies targeted EGFR and HER3 represent a particularly promising therapeutic avenue in TNBCBM, where effective CNS drug delivery remains a major obstacle [167,168,169]. Eventually, proteins may bridge the translational gap by being more readily implementable in clinical diagnostics than purely genomic, imaging, or histopathologic signatures. Clinically, this implicates earlier intervention tools in mitigating withstanding challenges in TNBCBM with its poor prognosis and limited treatments that effectively cross the blood–brain barrier.

It is important to present this data in the context of the strengths and limitations of our study. By examining clinicopathological and molecular biomarkers, we offer an integrative perspective that more holistically captures the clinical situation in which a physician may implement our findings. Amidst the various areas of development and innovation, our study provides a timely overview of current progression and also points to directions of future growth in biomarker discovery. Overall, we hope to have laid the groundwork for biomarker-driven clinical trials in TNBC aimed at identifying key pathways to prevent and/or treat BM. However, we also acknowledge that our findings are against the backdrop of a very heterogeneous tumor microenvironment, with certain findings that must be further stratified by subtype to be of greater value. Furthermore, there is a lack of consistent validation with a heterogeneous group of publications ranging from preclinical in vitro to retrospective cohort studies. Thus, standardization of findings and their comparative significance as well as formal study-level quality scoring remains challenging.

## 5. Conclusions

With a high propensity for brain metastasis, triple-negative breast cancer results in substantial morbidity and mortality with limited receptors that can be leveraged for treatment. Thus, biomarkers that predict metastasis to the CNS could allow for earlier risk stratification, tailored surveillance protocols, and more individualized and targeted treatment strategies. Our findings suggest that circulating protein biomarkers and tumor transcriptomic variability show particular promise as predictors of CNS metastatic potential. Nevertheless, the heterogeneity of TNBC underscores the continued need for multimodal approaches to early diagnosis and effective treatment. In particular, biomarkers have the potential to revolutionize clinical care for TNBC by changing the focus from treating brain metastases reactively to proactively monitoring and preventing them. Improving outcomes for this high-risk population will require bridging the gap between new research and clinical utility.

## Figures and Tables

**Table 1:** Characteristics of Included Studies by Study Type.

Study Type	N Studies	N Biomarkers	Control Group? % Yes; % No
Clinical [10,11,12,13,14,15,16,17,18,19,20,21,22,23,24,25,26,27,28,29,30,31,32,33,34,35,36,37,38,39,40,41,42,43,44,45,46,47,48,49,50,51,52,53,54,55,56,57,58,59,60,61,62,63,64,65,66,67,68,69,70]	61	93	73.6%; 26.4%
Pre-Clinical [71,72,73,74,75,76,77,78,79,80,81,82,83,84,85,86,87,88,89,90,91,92,93,94,95,96,97,98,99,100,101,102,103,104,105,106,107,108,109,110,111,112,113,114,115,116,117,118,119,120,121,122,123,124,125,126,127,128,129,130,131,132,133,134,135,136,137,138,139]	69	79	94.9%; 5.1%

**Table 2:** Pre-clinical studies analysis. The number of biomarkers (*n*) varies across columns because individual studies reported different biomarker-related variables. Specifically, not all studies provided numerical data for each biomarker and for each variable. For *n* = 2, the median value equals the mean, and for *n* = 1, the individual percentage was reported. N/A = not reported in any of the studies.

Category	Median % of TNBC Population with Biomarker ^1^	Median % of BM Within the TNBC Population that Has the Biomarker ^1^	% Primary; % Metastasis; % Both ^2^	Target for Treatment % Yes; % No ^3^
Clinical and Radiographic Features [71]	69.4% (*n* = 2)	N/A	100.0%; 0.0%; 0.0% (*n* = 2)	0.0%; 100.0% (*n* = 2)
Noncoding RNA [72,73,74,75,76,77,78,79]	49.2%; CI 27.0–100.0% (*n* = 3)	55.6%; CI 7.9–100.0% (*n* = 5)	100.0%; 0.0%; 0.0% (*n* = 10)	18.8%; 81.2% (*n* = 16)
Growth Factor Receptors [80,81,82,83,84,85,86,87,88,89,90]	72.2% (*n* = 1)	53.9% (*n* = 2)	72.7%; 18.2%; 9.1% (*n* = 11)	66.7%; 33.3% (*n* = 15)
Circulating Proteins [91,92,93,94,95,96,97,98]	N/A	100.0% (*n* = 1)	62.5%; 37.5%; 0.0% (*n* = 8)	37.5%; 62.5% (*n* = 8)
Transcriptomic Studies [99,100,101,102,103,104,105,106,107,108,109,110]	9.0%; CI 8.7–24.5% (*n* = 9)	64.0%; CI 50.9–87.7% (*n* = 7)	89.5%; 10.5%; 0.0% (*n* = 19)	47.8%; 52.2% (*n* = 23)
Cellular Signaling and Transport [111,112,113,114,115,116,117,118,119]	38.5% (*n* = 2)	63.0% (*n* = 1)	71.4%; 14.3%; 14.3% (*n* = 7)	87.5%; 12.5% (*n* = 8)
Immune Microenvironment Marker [120,121,122,123,124,125]	13.1% (*n* = 1)	70.0% (*n* = 1)	88.9%; 11.1%; 0.0% (*n* = 9)	60.0%; 40.0% (*n* = 10)
Hormone Signaling [129,130,131,132,133]	100.0% (*n* = 1)	83.3% (*n* = 1)	100.0%; 0.0%; 0.0% (*n* = 5)	75.0%; 25.0% (*n* = 4)
Metabolism and Stress Signaling [134,135,136,137,138,139]	73.0% (*n* = 1)	N/A	66.7%; 33.3%; 0.0% (*n* = 6)	45.5%; 54.5% (*n* = 11)

^1^ *n* = the number of biomarkers that reported percentage values for the proportion of biomarker positivity in TNBC and those who developed brain metastasis. ^2^ *n* = the total number of biomarkers that reported whether the biomarker was present in the primary tumor, metastatic lesion, or both, with percentages based on that total. ^3^ *n* = the number of biomarkers identified as targetable by existing or investigational therapies and those not currently considered targetable.

**Table 3:** Clinical studies analysis. The number of biomarkers (*n*) varies across columns because individual studies reported different biomarker-related variables. Specifically, not all studies provided numerical data for each biomarker and for each variable. For *n* = 2, the median value equals the mean, and for *n* = 1, the individual percentage was reported. N/A = not reported in any of the studies.

Category	Median % of TNBC Population with Biomarker ^1^	Median % of BM Within the TNBC Population that Has the Biomarker ^1^	% Primary; % Metastasis; % Both ^2^	Target for Treatment % Yes; % No ^3^
Clinical and Radiographic Features [10,11,12,13]	N/A	76.2% (*n* = 1)	75.0%; 25.0%; 0.0% (*n* = 4)	0.0%; 100.0% (*n* = 3)
Growth Factor Receptors [14,15,16,17,18,19,20,21]	33.0%; CI 16.2–75.0% (*n* = 5)	75.0%; CI 71.0–86.0% (*n* = 5)	14.3%; 85.7%; 0.0% (*n* = 7)	66.7%; 33.3% (*n* = 9)
Circulating Proteins [22,23,24,25]	70.0% (*n* = 1)	N/A	50.0%; 50.0%; 0.0% (*n* = 4)	75.0%; 25.0% (*n* = 4)
Transcriptomic Studies [12,13,14,15,16,17,18,19,20,21,22,23,24,25,26,27,28,29,30,31,32,33,34,35,36,37,38,39,40,41,42,43,44,45,46,47,48,49]	25.0%; CI 15.5–38.0% (*n* = 29)	37.5%; CI 16.0–56.3% (*n* = 29)	63.8%; 14.9%; 21.3% (*n* = 47)	58.3%; 41.7% (*n* = 24)
Cellular Signaling and Transport [47,50,51,52,53]	67.5% (*n* = 2)	57.0% (*n* = 2)	66.7%; 33.3%; 0.0% (*n* = 6)	66.7%; 33.3% (*n* = 6)
Immune Microenvironment Marker [54,55,56,57,58,59,60]	34.2% (*n* = 2)	76.0%; CI 50.0–90.0% (*n* = 6)	36.4%; 45.5%; 18.2% (*n* = 11)	54.5%; 45.5% (*n* = 11)
Hormone Signaling [61,62,63]	30.0% (*n* = 2)	65.0% (*n* = 2)	50.0%; 50.0%; 0.0% (*n* = 2)	83.3%; 16.7% (*n* = 6)
Metabolism and Stress Signaling [64,65,66,67,68,69,70]	48.5%; CI 32.0–73.0% (*n* = 4)	65.1%; CI 65.0–100.0% (*n* = 3)	37.5%; 62.5%; 0.0% (*n* = 8)	57.1%; 42.9% (*n* = 7)

^1^ *n* = the number of biomarkers that reported percentage values for the proportion of biomarker positivity in TNBC and those who developed brain metastasis. ^2^ *n* = the total number of biomarkers that reported whether the biomarker was present in the primary tumor, metastatic lesion, or both, with percentages based on that total. ^3^ *n* = the number of biomarkers identified as targetable by existing or investigational therapies and those not currently considered targetable.

## Data Availability

The raw data supporting the conclusions of this article will be made available by the authors on request.

## Supplementary Materials

The following supporting information can be downloaded at: https://www.mdpi.com/article/10.3390/ijms27041909/s1.

## Abbreviations

The following abbreviations are used in this manuscript:

TNBC	Triple Negative Breast Cancer
TNBCBM	Triple Negative Breast Cancer Brain Metastasis
BM	Brain Metastasis
CNS	Central Nervous System
ER	Estrogen Receptor
PR	Progesterone Receptor
HER2	Human Epidermal Growth Factor 2
miR	Micro RNA
EGFR	Epidermal Growth Factor Receptor
PRISMA	Preferred Reporting Items for Systematic Reviews and Meta-Analyses
MRI	Magnetic Resonance Imaging
CNR	Contrast-to-Noise Ratio
circKIF4A	Circular RNA Kinesin Family Member 4A
HER3	Human epidermal growth factor Receptor 3
c-MET	c-mesenchymal–epithelial transition factor
ShcD	Src homology and collagen D
PI3K	Phosphatidylinositol 3-kinase
Akt	Protein Kinase B
BBB	Blood–Brain Barrier
RTK	Receptor Tyrosine Kinase
MAPK	Mitogen-Activated Protein Kinase
PTEN	Phosphatase and Tensin Homolog
Shc	Src homology and collagen
CTC	Circulating Tumor Cell
EpCAM	Epithelial Cell Adhesion Molecule
ANGPTL4	Angiopoietin-like 4
SERPINB1	Serpin Family B Member 1
HYAL1	Hyaluronidase 1
CD24	cluster of differentiation 24
AnxA2	Annexin A2
PTHrP	Parathyroid Hormone-related Protein
ARID1A	AT-rich interaction domain 1A
SWI/SNF	Switch/Sucrose Non-Fermentable
TP53	Tumor Protein 53
BRCA2	Breast Cancer gene 2
ADAM8	A Disintegrin And Metalloproteinase domain 8
GATA3	GATA-binding protein 3
SOX-10	SRY-box transcription factor 10
PD-1	Programmed Cell Death Protein-1
PD-L1	Programmed Death-Ligand 1
FDA	Food and Drug Administration
CD8^+^	cluster of differentiation 8 positive
OS	Overall Survival
CNS-PFS	Central Nervous System-Progression Free Survival
PKC-θ	Protein Kinase C θ
mPRα	Membrane Progesterone Receptor α
IL-1β	interleukin-1β
PECAM	Platelet Endothelial Cell Adhesion Molecule
GRP94	Glucose-Regulated Protein 94
DDX3	Dead-box helicase 3
HIF1α	Hypoxia Inducible Factor 1-α
CI	Confidence Interval
