# Supplementary material for: Molecular and Clinicopathological Biomarkers Predicting Brain Metastasis in Triple-Negative Breast Cancer: A Systematic Review
Source: Int J Mol Sci. 2026 Feb 16;27(4):1909. doi: 10.3390/ijms27041909 (PMC12940659; doi:10.3390/ijms27041909)
Supplement: Supplementary file 1 [file ijms-27-01909-s001.zip › TNBCBM Review Manuscript_Table S1.pdf]

Supplemental Table S1. Biomarker Characteristics

Category of Biomarker	Included Studies
Clinical & Radiographic Features	Gabani 2019 [10]<br>Lin 2020 [11]<br>He 2024 [12]<br>Hamilton 2017 [71]<br>Cheng 2021 [13]
Noncoding RNA	Debeb 2015 [72]<br>Hammash 2022[73]<br>Pan 2021[74]<br>Figueira 2021[75]<br>Harati 2021[76]<br>Sereno 2020[77]<br>Wu 2024[78]<br>Xie 2025[79]
Growth Factor Receptors	Kusuhara 2022 [14]<br>Choi 2013 [80]<br>Wu 2019 [15]<br>Kalita-deCroft 2020 [16]<br>Subham 2023 [17]<br>Marquez-Ortiz 2021 [81]<br>Liu 2023 [82]<br>Thies 2018 [83]<br>Galloni 2024 [84]<br>Hohensee 2013 [18]<br>Alhusban 2021 [19]<br>Gong 2019 [85]<br>Leontovich 2018 [86]<br>Lau 2021 [87]<br>Adkins 2015 [88]<br>Masiero 2019 [89]<br>Wang 2020 [20]<br>Lau 2025 [90]<br>Martinez-Aranda 2015 [21]
Circulating Proteins	Qiu 2025 [91]<br>Castro 2020 [22]<br>Avraham 2014 [92]<br>Carvalho 2024 [93]<br>Simeon 2021 [94]<br>Molnar 2020 [95]<br>Riebensahm 2019 [23]<br>Maji 2015 [24]<br>Hebert 2020 [96]<br>Cicero 2023 [97]

	Li 2023 [98]<br>Witzel 2017 [25]
Transcriptomic Studies	Manogna 2024 [26]<br>LoNigro 2012 [27]<br>Kabraji 2020 [28]<br>Klimov 2017 [29]<br>Morikawa 2019 [30]<br>HuangRSP 2021 [31]<br>Moreno 2023 [32]<br>Vidula 2025 [33]<br>Wikman 2012 [34]<br>Giannoudis 2021 [35]<br>Huang 2021 [36]<br>Galego 2021 [37]<br>Gan 2024 [99]<br>Echeverria 2018 [100]<br>Qian 2017 [101]<br>Ren 2018 [102]<br>Seehawer 2024 [103]<br>Shen 2022 [104]<br>Sun 2022 [105]<br>Woditschka 2012 [106]<br>Hamester 2022 [38]<br>Gonzalez-Angulo 2011 [39]<br>Adamo 2011 [40]<br>Hohensee 2017 [41]<br>He 2025 [42]<br>DeLara 2019 [43]<br>Statz 2021 [44]<br>Peluffo 2019 [107]<br>Sirkisoon 2020 [108]<br>Thulin 2021 [45]<br>Rong 2020 [46]<br>Dionisio 2020 [109]<br>RojasL 2019 [47]<br>Dahn 2022 [110]<br>Yamashita 2020 [48]<br>He 2024 [12]<br>Xiu 2016 [49]
Cellular Signaling and Transport	Assaker 2020 [50]<br>Reimer 2023 [51]<br>Gupta 2021 [111]<br>Hamester 2022 [112]<br>Ranjan 2016 [113]<br>Arumugan 2021 [114]<br>Vaidya 2020 [115]

	Choy 2016 [52]<br>Cerbelli 2023 [53]<br>Sayyad 2019 [116]<br>RojasL 2019 [47]<br>Romagnoli 2014 [117]<br>Fontana 2023 [118]<br>Dai 2022 [119]
Immune microenvironment markers	Shi 2025 [54]<br>Cao 2020 [55]<br>Cheng 2021[56]<br>Cimino-Mathews 2013 [57]<br>Joshi 2024 [58]<br>Sambade 2019 [59]<br>Motallebnejad 2022 [120]<br>Lyle 2016 [121]<br>Meszarosa 2023 [122]<br>Llevenes 2024 [123]<br>Kim 2018 [124]<br>Stirling 2022 [125]<br>Loree 2021[60]<br>Gourgue 2020 [126]<br>Foo 2022 [127]<br>Dunn 2022 [128]<br>Xiu 2016 [49]
Hormone signaling	Kaider-Person 2018 [61]<br>Cittelly 2015 [129]<br>Contreras-Zarate 2019 [130]<br>Sanchez-Juarez 2025 [131]<br>Sartorius 2016 [132]<br>Bergen 2021 [62]<br>Zhou 2017 [133]<br>Jiaxin 2022 [63]
Metabolism and Stress Signaling	Maric 2023 [134]<br>Martinez-Aranda 2015 [41]<br>Voduc 2015 [64]<br>Jung 2015 [65]<br>Ebright 2020 [66]<br>Butler 2020 [67]<br>Malin 2014 [135]<br>Adamo 2011 [136]<br>Santana-Codina 2020 [68]<br>Serhan 2024 [137]<br>Heerma van Voss 2017 [138]<br>BlackmanMCNM 2022 [69]<br>Vogel-Gonzalez 2021 [139]

	Hashemi-Sadraei 2018 [70]
