# Supplementary material for: Molecular and Clinicopathological Biomarkers Predicting Brain Metastasis in Triple-Negative Breast Cancer: A Systematic Review
Source: Int J Mol Sci. 2026 Feb 16;27(4):1909. doi: 10.3390/ijms27041909 (PMC12940659; doi:10.3390/ijms27041909)
Supplement: Supplementary file 1 [file ijms-27-01909-s001.zip › TNBCBM Review Manuscript_Table S2.pdf]

Supplemental Table S2. Search Criteria Specific to Each Database

Database	Search String
Scopus	ALL (triple negative breast cancer OR "TNBC") AND ALL ("brain* metastasis" OR "CNS metastasis*" OR "neurologic dissemination*") AND ALL(“predictive* biomarker” OR “clinicopathological mechanism*”) AND PUBYEAR > 2009 AND PUBYEAR < 2026 AND (LIMIT-TO (DOCTYPE , "ar") OR LIMIT-TO (DOCTYPE , "cp") OR LIMIT-TO (DOCTYPE , "dp")) AND (LIMIT-TO (LANGUAGE , "English")) AND (EXCLUDE (EXACTKEYWORD , "Nonhuman") OR EXCLUDE (EXACTKEYWORD , "Mouse") OR EXCLUDE (EXACTKEYWORD , "Animals") OR EXCLUDE (EXACTKEYWORD , "Animal") OR EXCLUDE (EXACTKEYWORD , "Animal Experiment") OR EXCLUDE (EXACTKEYWORD , "Animal Model") OR EXCLUDE (EXACTKEYWORD , "Mice") OR EXCLUDE (EXACTKEYWORD , "Animal Tissue") OR EXCLUDE (EXACTKEYWORD , "Animal Cell") OR EXCLUDE (EXACTKEYWORD , "Mice, Nude") OR EXCLUDE (EXACTKEYWORD , "Rat"))
Web of Science	((triple negative breast cancer OR "triple negative breast cancer" OR TNBC) AND ("brain metastasis" OR "CNS metastasis" OR "metastatic brain lesions" OR "brain colonization" OR "neurologic dissemination"))
Cochrane Library	((triple negative breast cancer OR "triple negative breast cancer" OR TNBC) AND ("brain metastasis" OR "CNS metastasis" OR "neurologic dissemination" OR "metastatic brain* lesions" OR "brain* colonization"))
PubMed	(triple negative breast cancer OR TNBC) AND ("brain* metastasis" OR "metastatic brain* lesions" OR "brain* colonization" OR "CNS metastasis*" OR "neurologic* dissemination")
Embase	(('triple negative breast cancer'/exp OR triple) AND negative AND breast AND cancer OR tnbc) AND ('brain* metastasis' OR 'metastatic brain* lesions' OR 'brain* colonization' OR 'cns metastasis' OR 'neurologic dissemination*')
