# Supplementary material for: Molecular and Clinicopathological Biomarkers Predicting Brain Metastasis in Triple-Negative Breast Cancer: A Systematic Review
Source: Int J Mol Sci. 2026 Feb 16;27(4):1909. doi: 10.3390/ijms27041909 (PMC12940659; doi:10.3390/ijms27041909)
Supplement: Supplementary file 1 [file ijms-27-01909-s001.zip › TNBCBM Review Manuscript_Table S3.pdf]

Supplemental Table S3. Full List of Actionable Biomarkers Identified, Associated Approved Therapeutic Interventions, and Relevant Ongoing Relevant Clinical Trials

Name of Biomarker	Examples of Associated Therapies / Approved Drugs	Examples of Ongoing / Current Clinical Trials
3q Gene Abnormalities	Azacitidine	N/A [170]
FASN	- Denifanstat (TVB-2640)<br>- TVB-3166 and TVB-3664	- NCT02223247: Phase I, TVB-2640 monotherapy and with paclitaxel in advanced solid tumors - COMPLETED.<br>- Phase II: TVB-2640 + bevacizumab in recurrent high-grade astrocytoma - COMPLETED.<br>- Omeprazole – Inhibiting Fatty Acid Synthase with Omeprazole to Improve Efficacy of Neoadjuvant Chemotherapy in Patients with Operable TNBC<br>[171 - 173]
HMGCR	- Statins<br>- PROTAC-based HMGCR degraders	- Window-of-opportunity trial: High-dose atorvastatin (80 mg/day for 2 weeks) in 50 breast cancer patients - COMPLETED.<br>- Window-of-opportunity trial: Atorvastatin (80 mg/day) in 42 breast cancer patients - COMPLETED.<br>- Window-of-opportunity trial: Simvastatin in early-stage breast cancer - COMPLETED.<br>[174-176]
CPT1A	- Etomoxir: Irreversible CPT1 inhibitor (withdrawn from clinical development due to hepatotoxicity and off-target effects)<br>- ST1326: CPT1A-selective inhibitor (preclinical only)<br>- DHP-B (2,6-dihydroxypeperomin B): Novel covalent CPT1A inhibitor (preclinical only)<br>- CPI-613 (devimistat)	N/A
ADAM8	- BK-1361 Peptidomimetic ADAM8 inhibitor targeting the disintegrin domain, prevents ADAM8 multimerization (preclinical only)<br>- Dimeric arylsulfonamides (compound 8): Bivalent ADAM8 inhibitor selective over ADAM10 and MMPs, inhibits at nanomolar concentration (preclinical only)<br>- Adam8-Apt1-26nt: RNA aptamer targeting soluble ADAM8 metalloproteinase domain (preclinical only)	N/A

AKT1	- Capivasertib (AZD5363): ATP-competitive pan-AKT inhibitor; FDA-approved (2023) in combination with fulvestrant for HR+/HER2- breast cancer with PIK3CA, AKT1, or PTEN alterations	- LOTUS trial (NCT02162719): Phase II trial of ipatasertib + paclitaxel vs. placebo + paclitaxel as first-line therapy for mTNBC - COMPLETED.<br>- FAIRLANE trial: Phase II neoadjuvant ipatasertib + paclitaxel in early TNBC - COMPLETED.<br>- IPATunity130 (NCT03337724): Phase III trial of ipatasertib + paclitaxel in mTNBC - COMPLETED.<br>[177 - 179]
ERBB2	- Trastuzumab (Herceptin)<br>- Trastuzumab and hyaluronidase-oysk (Herceptin Hylecta)<br>- Pertuzumab (Perjeta)	- SUMMIT (NCT01953926): Phase II basket trial of neratinib ± trastuzumab in HER2-mutant cancers - COMPLETED.<br>[180]
ESR1	- Elacestrant (Orserdu): First oral SERD approved by FDA (January 2023) for ER+/HER2-/ESR1-mutant advanced or metastatic breast cancer after progression on at least one line of endocrine therapy including a CDK4/6 inhibitor<br>- Imlunestrant: Oral SERD approved for ER+/HER2-/ESR1-mutant breast cancer after progression on aromatase inhibitor ± CDK4/6 inhibitor	N/A
PIK3CA	- Alpelisib (Piqray): α-selective PI3K inhibitor and degrader; FDA-approved (May 2019) in combination with fulvestrant for HR+/HER2-/PIK3CA-mutant advanced breast cancer after progression on endocrine therapy<br>- Inavolisib: Highly selective PI3Kα inhibitor with mutant-specific degradation; FDA-approved in combination with palbociclib + fulvestrant for HR+/HER2-/PIK3CA-mutant advanced breast cancer with early relapse on adjuvant endocrine therapy	- NCT01790932/NCT01629615: Phase II trial of buparlisib monotherapy in metastatic TNBC - COMPLETED.<br>- Gedatolisib + cofetuzumab pelidotin phase I: Phase I trial in metastatic TNBC - COMPLETED.<br>- NCT04345913: Phase I/II trial of Eribulin + copanlisib in metastatic TNBC - ONGOING<br>[181 - 183]
Androgen Receptor	- AR antagonists<br>- Abiraterone acetate (Zytiga)	- NCT01889238: Phase II trial of enzalutamide in AR+ TNBC - COMPLETED.<br>- UCBG 3-06 START: Phase II trial of darolutamide vs. capecitabine in AR+ advanced TNBC - COMPLETED.<br>- NCT06099769: Phase II randomized trial of enzalutamide vs. enzalutamide + mifepristone (GR antagonist) vs. physician's choice chemotherapy in AR+ metastatic TNBC - ONGOING.<br>[184 - 186]
Angiopoietin-2 (ANGPT2)	• BI 836880 (investigational; anti-ANGPT2 monoclonal antibody)	• NCT05249426: BI 836880–based immunotherapy combination in solid tumors — Active, not recruiting

ANGPTL4	• MAR001 (Anti-ANGPTL4 monoclonal antibody; investigational; non-oncology development reported)	N/A
TGF-β2 (TGFB2)	• Trabedersen / OT-101 (TGFB2 antisense) (investigational)<br>• Vactosertib / TEW-7197 (TGF-βRI/ALK5 inhibitor; TGF-β pathway) (investigational)<br>• Galunisertib / LY2157299 (TGF-βRI/ALK5 inhibitor; TGF-β pathway) (investigational)<br>• Bintrafusp alfa / M7824 (PD-L1×TGF-β trap; TGF-β pathway) (investigational)<br>• Ficerafusp alfa / BCA101 (EGFR×TGF-β inhibitor; TGF-β pathway) (investigational)<br>• Fresolimumab / GC1008 (pan–TGF-β neutralizing mAb; includes TGF-β2) (investigational)	• NCT06579196: OT-101 (trabedersen) + pembrolizumab – 1L PD-L1+ advanced/metastatic NSCLC<br>• NCT06079346: OT-101 + mFOLFIRINOX – advanced unresectable/metastatic pancreatic cancer<br>• NCT06788990: Ficerafusp alfa (BCA101) + pembrolizumab vs placebo + pembrolizumab – 1L PD-L1+ recurrent/metastatic HNSCC<br>• NCT05588648: Vactosertib – recurrent/refractory/progressive osteosarcoma
Annexin-A1 (ANXA1)	N/A	- ATTAINMENT (ISRCTN78740398): Phase Ib first-in-human trial of MDX-124 alone and in combination with anti-cancer treatments in advanced solid tumors - ONGOING.<br>[187]
AXL/GAS6	N/A	- NCT03639246: Phase Ib trial of batiraxcept + paclitaxel or PLD in platinum-resistant ovarian cancer - COMPLETED.<br>- NCT04300140: Phase Ib/2 trial of batiraxcept + cabozantinib in previously treated ccRCC - ONGOING.<br>[188]
B2 adrenergic receptor (ADRB2)	• Beta-blockers (approved; repurposed)	• NCT05741164: Propranolol + pembrolizumab in checkpoint-inhibitor–refractory metastatic/unresectable TNBC — Recruiting<br>[189]
B7-H3 (CD276)	N/A	- NCT01391143: Phase I, dose escalation study of MGA271 (Fc-optimized humanized anti-B7-H3 monoclonal antibody) in patients with solid tumors (including TNBC)<br>- NCT03729596: Phase 1/2, vobramitamab duocarmazine (Anti-B7-H3 Antibody Drug Conjugate) in patients with solid tumors (including TNBC)<br>[190, 191]

BRCA 1 and 2	- Olaparib (Lynparza)<br>- Talazoparib (Talzenna)<br>- Fuzuloparib<br>- Carboplatin and Cisplatin	- OlympiA (NCT02032823): Phase III trial of adjuvant olaparib vs. placebo in high-risk HER2-negative early breast cancer with germline BRCA1/2 mutation - COMPLETED.<br>- PARTNER (NCT03150576): Phase II/III trial of neoadjuvant carboplatin/paclitaxel ± olaparib in TNBC and/or germline BRCA breast cancer - ONGOING.<br>[192, 193]
CD44	• CD44-targeted CAR-T / bispecific CAR-T approaches (investigational)	• NCT05577091: CD44/CD133 dual-target CAR-T (Tris-CAR-T) for recurrent glioblastoma — Active, not recruiting<br>[194]
EpCAM	N/A	- NCT00513292: Open-label, phase II, randomized trial of adecatumumab monotherapy (2 mg/kg vs. 6 mg/kg q2w) in metastatic breast cancer - COMPLETED.<br>- NCT05028933 (CT03/CT04): Phase I first-in-human trial of IMC001 EpCAM CAR-T cells in advanced GI cancers - ONGOING.<br>[195, 196]
P-cadherin	N/A	- NCT05957471: Phase I first-in-human trial of BC3195 in advanced solid malignancies - ONGOING.<br>- NCT02375958: Phase I trial of PCA062 in solid tumors - COMPLETED/TERMINATED.<br>[197, 198]
c-Met (MET)	• Capmatinib (Tabrecta)<br>• Tepotinib (Tepmetko)<br>• Crizotinib (investigational for MET)<br>• Savolitinib (investigational)<br>• Gumarontinib/Glumetinib (investigational)<br>• Cabozantinib (investigational for MET)<br>• Amivantamab (EGFR/MET bispecific; approved in NSCLC contexts)<br>• Sym015 (anti-MET mAb mixture) (investigational)	• NCT04270591: Glumetinib (gumarontinib) – c-MET-positive NSCLC<br>• NCT05261399: Savolitinib + osimertinib vs platinum doublet (SAFFRON) – EGFR-mut with MET overexpression/amplification NSCLC<br>[199, 200]
c-Met/B1 integrin complex	N/A	- NCT04608812: OS2966 (anti-Beta1 Integrin) antibody, Phase 1, in high grade glioma<br>- CCTG IND197 (NCT01147484): Phase II trial of foretinib in metastatic TNBC - COMPLETED.<br>- Cabozantinib phase II: Phase II trial in metastatic TNBC - COMPLETED.<br>- NCT06084481: Phase Ib signal-seeking study of ABBV-400 (c-Met ADC) in advanced solid tumors including TNBC (n=20) and HR+/HER2- breast cancer (n=20) - ONGOING.<br>[201 - 204]

CD44	N/A	- NCT01358903: Phase I trial in advanced CD44-expressing solid tumors - COMPLETED.<br>- NCT05725291: Phase I first-in-human trial in advanced solid tumors - ONGOING.<br>[205, 206]
CXCL8	N/A	- Phase I trial of SBRT + nivolumab + BMS-986253 in advanced solid tumors, melanoma, and RCC - ONGOING.<br>- NCT04572451: Phase I trial of SBRT + nivolumab + BMS-986253 in advanced solid tumors, melanoma, and RCC - ONGOING.<br>- NCT03400332: Phase I/II trial of BMS-986253 + nivolumab in advanced cancers - COMPLETED.<br>- NCT02370238: Phase II trial of reparixin + paclitaxel in metastatic TNBC - COMPLETED.<br>- NCT01861054: Phase Ib pilot study of reparixin in early breast cancer - COMPLETED.<br>[207 - 209]
E2	- Tamoxifen<br>- Toremifene<br>- Raloxifene<br>- Fulvestrant	- NCT01194427 (Wisconsin Oncology Network): Phase 2 trial of high-dose estradiol (10 mg TID) in metastatic TNBC targeting ERβ - COMPLETED.<br>- SERENA-2 (NCT04214288): Phase 2 trial of camizestrant vs. fulvestrant in ER-positive, HER2-negative advanced breast cancer - COMPLETED.<br>- SERENA-6: Phase 3 trial of camizestrant in ER-positive, HER2-negative advanced breast cancer - ONGOING<br>[210, 211]
EDB-FN	• PYX-201 (investigational; EDB-FN–targeting ADC)	• NCT05720117: PYX-201 (EDB-FN–targeting ADC) in advanced solid tumors — Recruiting<br>[212]
EGFR	• EGFR inhibitors (approved): erlotinib; gefitinib; afatinib; osimertinib; cetuximab; panitumumab; necitumumab; amivantamab; mobocertinib (EGFR exon20)<br>• EGFR inhibitors (investigational/CNS-penetrant): WSD0922-FU	• NCT04197934: WSD0922-FU (CNS-penetrant EGFR inhibitor) — glioblastoma / anaplastic astrocytoma / NSCLC with CNS metastases<br>[213]
PTEN	• PI3K/AKT pathway (PTEN-altered; approved): capivasertib + fulvestrant; HR+/HER2- breast cancer with PIK3CA/AKT1/PTEN alterations)	• NCT03218826: AZD8186 + docetaxel (PI3Kβ inhibitor strategy for PTEN/PIK3CB-altered tumors) — advanced solid tumors<br>[214]
RAC1	N/A	- R-Ketorolac, targets Rac1 in ovarian cancer<br>[215]

ARID1A	N/A	- NCT06617923: Phase 2, Senaparib with TMX for ARID1A mutation associated ovarian cancer<br>NCT05490472: Phase 2, JAB-2485, patientes with ARID1A mutant solid tumors (inlcuding TNBC)<br>[216]
CDH1	N/A	NCT03620643: Crizotinib, phase 2, in CDH1 mutated solid tumors (including TNBC)<br>[217]
PECAM1	- Anti-PECAM-1 monoclonal antibodies (preclinical)	N/A
FN14 (TNFRSF12A)	• Enavatuzumab (PDL192/ABT-361; anti-Fn14 mAb; investigational)<br>• RG7212 (anti-TWEAK mAb; investigational)	N/A
GRP94 (HSP90B1/gp96)	• HSPPC-96 (autologous gp96 heat shock protein–peptide complex vaccine; investigational)	N/A
GFAP	- GFAP antisense oligonucleotides (ASOs) (clinical development)<br>- Anti-GFAP monoclonal antibody (preclinical)	- NCT04849741 (Ionis Pharmaceuticals): Phase 1/2 study of ION373, a GFAP-targeting antisense oligonucleotide, in patients with Alexander disease - ONGOING.<br>[218]
HAS2	- 4-Methylumbelliferone (4-MU / Hymecromone) (FDA-approved as choleretic agent; repurposing for oncology)<br>- DDIT (5'-Deoxy-5'-(1,3-Diphenyl-2-Imidazolidinyl)-Thymidine) (preclinical)<br>- Compound VII (10'-methyl-6'-phenyl-3'H-spiro[piperidine-4,2'-pyrano[3,2-g]chromene]-4',8'-dione) (preclinical)	- NCT00225537 (Phase 1): 4-Methylumbelliferone (hymecromone) safety study in healthy volunteers - COMPLETED.<br>[219]
HER3 (ERBB3)	• Patritumab deruxtecan (HER3-DXd; U3-1402/MK-1022) — investigational HER3-targeted ADC<br>• Seribantumab (MM-121) — investigational anti-HER3 mAb<br>• HMBD-001 — investigational anti-HER3 mAb<br>• Zenocutuzumab (MCLA-128) — investigational HER2×HER3 bispecific Ab	• NCT06797635: Patritumab deruxtecan (MK-1022/HER3-DXd) + pembrolizumab ± other agents — neoadjuvant approach in high-risk early-stage TNBC / HR-low HER2- breast cancer<br>• NCT06596694: Patritumab deruxtecan (MK-1022/HER3-DXd) — advanced GI cancers<br>[220, 221]
HER4 (ERBB4)	• Afatinib (approved; pan-HER TKI incl. HER4)<br>• Neratinib (approved; pan-HER TKI incl. HER4)	• NCT04872985: Pyrotinib + neoadjuvant chemotherapy — HR+/HER2-, HER4 high-expression breast cancer<br>[222]

	• Dacomitinib (approved; pan-HER TKI incl. HER4)<br>• Pyrotinib (pan-ErbB TKI incl. HER4; investigational/non-US approval)	
	- Belzutifan (Welireg) (FDA-approved)<br>- Acriflavine (clinical development)<br>- Minnelide (clinical development)	- NCT00466583: Phase 1 pilot trial of EZN-2968 (HIF-1α antisense oligonucleotide) in patients with refractory solid tumors - COMPLETED.<br>- NCT01120288: Phase 1 trial of EZN-2968 in patients with advanced solid tumors with liver metastases - COMPLETED<br>- NCT00422721: Phase 1 trial of 2-Methoxyestradiol (Panzem) in patients with advanced solid tumors - COMPLETED<br>[223, 224]
HYAL1	- Sulfated Hyaluronic Acid (sHA) (preclinical)<br>- Delphinidin (preclinical; natural compound found in fruits/vegetables)	- N/A
	- IL13Rα2-targeted CAR-T cells (clinical development)<br>- IL13-PE38QQR (Cintredekin besudotox) (clinical development - Phase 3 completed)<br>- Anti-IL13Rα2 monoclonal antibodies (mAb15D8) (preclinical)	- NCT02208362: Phase 1 trial of IL-13Rα2-targeting CAR-T cells in recurrent high-grade glioma - COMPLETED.<br>- NCT00730613: Phase 1 pilot safety and feasibility trial of IL-13Rα2-targeted CAR-T cells in recurrent GBM - COMPLETED.<br>- Phase 3 trial of IL13-PE38QQR (Cintredekin besudotox) in glioblastoma - COMPLETED.<br>[225-227]
Integrin Signaling Axis	• Sigvotatug vedotin / SGN-B6A (investigational; ITGB6-targeted ADC) ;<br>• PF-08046876 (investigational; integrin-targeted mAb)	• NCT04389632: Sigvotatug vedotin (SGN-B6A; ITGB6-targeted ADC) in advanced solid tumors — Active, not recruiting<br>• NCT07090499: PF-08046876 (integrin-targeted mAb) in advanced solid tumors — Recruiting<br>[228, 229]
Integrin β1, FAK	• Defactinib (investigational; FAK inhibitor)	• NCT06194929: Defactinib + pembrolizumab for brain metastases from melanoma — Active, recruiting<br>• NCT05512208: Defactinib-based regimen in solid tumors (gynecologic) — Active, recruiting<br>[230, 231]
ISG15	- Hyperoside (HYP) (preclinical)<br>- USP18 siRNA/shRNA/CRISPR knockout (preclinical)	N/A
THBS1	• VT1021 (investigational; thrombospondin-1/TSP-1 activator)	• NCT03970447: VT1021 within GBM AGILE platform (glioblastoma) — Active, recruiting<br>[232]

JAG1	- CTX014 (clinical development): Humanized anti-Jagged1/2-blocking antibody)<br>- Anti-JAG1 neutralizing mAbs	- NCT01695005: Phase 1 first-in-human study of LY3039478 (oral Notch inhibitor) in advanced or metastatic cancer - COMPLETED.<br>- Multiple Phase 1/2 trials of gamma-secretase inhibitors (RO4929097, MK-0752, PF-03084014) in solid tumors including breast cancer - COMPLETED/ONGOING<br>[233, 234]
LC3B and FIP200/Atg17	- FYCO1 peptide analogs (preclinical)<br>- Stapled peptide inhibitors of LC3B (preclinical)	- NCT04214418 (MEKiAUTO): Phase 1/2 trial of cobimetinib (MEK inhibitor) + atezolizumab (anti-PD-L1) + hydroxychloroquine (autophagy inhibitor) in KRAS-mutant pancreatic and colorectal cancer - ONGOING<br>- Phase 2 trial of GNS561 + atezolizumab + bevacizumab in hepatocellular carcinoma - ONGOING<br>[235, 236]
MAP2K4	- HRX-0233 (clinical development)<br>- PLX8725 (preclinical - Phase 1 warranted)<br>- BSJ-04-122 (preclinical)	- NCT04534283: Phase 2 basket trial of LY3214996 (ERK1/2 inhibitor) + abemaciclib (CDK4/6 inhibitor) in patients with tumors harboring pathogenic alterations in BRAF, RAF1, MAP2K1/2, ERK1/2, and NF1 - ONGOING.<br>- Phase 1 trials of PLX8725 in uterine leiomyosarcoma with MAP2K4 amplification - WARRANTED<br>[237, 238]
NCOR1	- CIM7 (RARα/NCoR1 complex disruptor) (preclinical)	N/A
COX-2 gene expression	- NSAIDs	- NCT01150045 (CALGB/SWOG 80702): Phase 3 randomized trial of celecoxib vs placebo added to standard adjuvant FOLFOX chemotherapy in stage III colon cancer - COMPLETED.<br>- REACT Trial (ISRCTN10059974): Phase 3 randomized trial of celecoxib vs placebo as adjuvant therapy in primary breast cancer - COMPLETED.<br>- REMAGUS02 Trial (ISRCTN10059974): Phase 2 trial of celecoxib with neoadjuvant chemotherapy in HER2-negative breast cancer - COMPLETED.<br>- NCT01695226: Phase 2 randomized trial of pre-operative celecoxib in primary breast cancer - COMPLETED.<br>[239-242]
NOTCH3	N/A	- NCT02129205 (Phase I - completed): PF-06650808 (anti-NOTCH3 ADC) in patients with advanced breast cancer and other solid tumors<br>- NCT01277146 (Phase I - completed): Tarextumab (OMP-59R5) dose escalation and expansion in solid tumors

		- NCT01695005 (Phase I - completed): LY3039478 (oral GSI) first-in-human study in advanced/metastatic cancer<br>[243 - 245]
PARP1	- Olaparib (Lynparza) (FDA-approved): First-in-class PARP1/2 inhibitor; approved for germline BRCA-mutated HER2-negative metastatic breast cancer (2018), adjuvant treatment of high-risk early breast cancer with gBRCA mutation (OlympiA trial, 2022); category 1 preferred for first-line and second-line TNBC with gBRCA1/2 mutation per NCCN guidelines<br>- Talazoparib (Talzenna) (FDA-approved): PARP1/2 inhibitor with highest PARP trapping potency; approved for germline BRCA-mutated HER2-negative locally advanced or metastatic breast cancer (EMBRACA trial); category 1 preferred for TNBC with gBRCA1/2 mutation per NCCN guidelines<br>- Rucaparib (Rubraca) (FDA-approved for ovarian/prostate cancer): PARP1/2/3 inhibitor; approved for BRCA-mutated ovarian cancer and prostate cancer; studied in breast cancer clinical trials	- NCT02000622 (OlympiAD) (Phase III - completed): Olaparib vs chemotherapy in germline BRCA-mutated HER2-negative metastatic breast cancer<br>- NCT01945775 (EMBRACA) (Phase III - completed): Talazoparib vs chemotherapy in germline BRCA-mutated HER2-negative advanced breast cancer<br>- NCT02032823 (OlympiA) (Phase III - completed): Adjuvant olaparib vs placebo in high-risk early breast cancer with gBRCA mutation<br>[246 - 248]
PD-L1	- Durvalumab (Imfinzi): Anti-PD-L1 antibody; FDA-approved for unresectable stage III NSCLC after chemoradiation (PACIFIC trial), extensive-stage SCLC, biliary tract cancer, hepatocellular carcinoma, endometrial cancer; in combination with tremelimumab for NSCLC and HCC<br>- Pembrolizumab (Keytruda): Anti-PD-1 antibody; FDA-approved for >20 cancer types including TNBC (with chemotherapy for PD-L1 CPS ≥10 metastatic disease; neoadjuvant/adjuvant for early-stage TNBC regardless of PD-L1), NSCLC, melanoma, head and neck cancer, gastric	- NCT02819518 (KEYNOTE-355) (Phase III - completed): Pembrolizumab + chemotherapy vs placebo + chemotherapy in first-line metastatic TNBC<br>- NCT05382286 (ASCENT-04/KEYNOTE-D19) (Phase III - completed): Sacituzumab govitecan + pembrolizumab vs chemotherapy + pembrolizumab in first-line PD-L1+ metastatic TNBC<br>- NCT03036488 (KEYNOTE-522) (Phase III - completed): Neoadjuvant pembrolizumab + chemotherapy followed by adjuvant pembrolizumab vs placebo in early-stage TNBC<br>[249 - 251]

	cancer, cervical cancer, MSI-H/dMMR tumors, TMB-H tumors; preferred first-line for PD-L1 CPS ≥10 metastatic TNBC per NCCN guidelines	
PDGFRB	- Imatinib (Gleevec): FDA-approved for CML, Ph+ ALL, GIST, dermatofibrosarcoma protuberans (DFSP), myeloid/lymphoid neoplasms with PDGFRB rearrangement; potent inhibitor of PDGFRB, BCR-ABL, c-KIT; standard of care for PDGFRB-rearranged myeloid neoplasms at 100 mg dail<br>- Sunitinib (Sutent): FDA-approved for RCC, GIST, pancreatic neuroendocrine tumors; targets PDGFRs, VEGFRs, c-KIT, RET, FLT3; inhibited TNBC cell viability, migration, and enhanced doxorubicin effects in preclinical studies<br>- Sorafenib (Nexavar): FDA-approved for RCC, HCC, differentiated thyroid cancer; targets RAF, PDGFRβ, VEGFR2, c-KIT, FLT3	- NCT01234337 (Phase I/II - completed): Pazopanib combinations in breast cancer [252]
Pericytes	• Ontuxizumab / MORAb-004 (anti-endosialin/TEM1/CD248; pericyte/perivascular-associated target; investigational)	N/A
IGF2	• Xentuzumab / BI 836845 (IGF-1/IGF-2 neutralizing mAb; investigational)<br>• Dusigitumab / MEDI-573 (IGF-1/IGF-2 neutralizing mAb; investigational)	N/A

PKC-theta	- Sotrastaurin (AEB071): Oral PKC inhibitor targeting multiple isoforms including PKCθ<br>- Enzastaurin (LY317615): PKCβ inhibitor with activity against other PKC isoforms<br>- Midostaurin (PKC-412, CGP 41251): Multikinase inhibitor targeting PKC isoforms and FLT3	- NCT01430416 (Phase I - completed): Sotrastaurin (AEB071) in metastatic uveal melanoma<br>- NCT01801358 (Phase Ib - completed): Sotrastaurin + binimetinib (MEK inhibitor) in metastatic uveal melanoma<br>- NCT01430416 (Phase Ib - completed): Sotrastaurin + alpelisib (PI3Kα inhibitor) in metastatic uveal melanoma<br>[253 - 255]
Progesterone Receptor a	- PR antagonists	- MIPRA Trial (NCT02651844): Phase II window-of-opportunity trial of mifepristone in PRA-high luminal breast cancer<br>NCT06099769: Phase II randomized trial of enzalutamide ± mifepristone vs. chemotherapy in AR+ metastatic TNBC<br>- Phase II window-of-opportunity trial of telapristone acetate in early-stage breast cancer<br>[186, 256, 257]
proNGF	• Anti-NGF monoclonal antibodies (pathway-level; investigational) — tanezumab; fulranumab	N/A
RAD51	N/A	- NCT03997968 (Phase I/II - Phase I completed, Phase II ongoing): CYT-0851 in advanced solid and hematologic cancers<br>[258]
RANK and RANKL (TNFRSF11A / TNFSF11)	• Denosumab (approved; anti-RANKL monoclonal antibody)	• NCT04711109: Denosumab vs placebo — breast cancer prevention in women with BRCA1 germline mutation (includes invasive TNBC endpoint)<br>• NCT04067726: Denosumab (RANKL inhibition) — mammographic breast density / breast cancer prevention biomarker setting<br>[259, 260]
RARRES2 (chemerin)	• CCX832 (CMKLR1/ChemR23 antagonist; investigational—development discontinued after Phase 1)	N/A
SIRPa	N/A	- NCT03990233: BI 765063 Phase I monotherapy in advanced solid tumors<br>- ASPEN-06 Phase II trial in gastric cancer

		- AO-176 Phase 1 trials (NCT03834948, NCT04445701): anti-CD47 antibody in combination studies<br>[261 - 263]
Syndecan-1 (SDC1/CD138)	• Indatuximab ravtansine / BT062 (investigational; anti-CD138 ADC) ; • CD138-directed CAR-T (investigational)	• NCT03672318: CAR138 T cells (CD138-directed CAR-T) in relapsed/refractory multiple myeloma<br>[264]
TFRC (transferrin receptor / CD71)	• CX-2029 (investigational; CD71-targeting Probody drug conjugate)	N/A
TP53	- Rezatapopt (PC14586) (not FDA approved)<br>Note: Mutation-specific p53 reactivator targeting Y220C mutant; currently in Phase II registrational trial (NCT04585750) for advanced solid tumors harboring TP53 Y220C mutation; showed favorable safety profile and preliminary efficacy in Phase I<br>- COTI-2 (not FDA approved)<br>Note: Third-generation thiosemicarbazone; reactivates mutant p53; Phase I trial in advanced gynecological cancers; preclinical studies showed anticancer activity in mutant p53-expressing models	- NCT03072043: Phase II trial of eprenetapopt plus azacitidine in TP53-mutant MDS/AML<br>- NCT04214860: Phase I/II trial of eprenetapopt plus venetoclax and azacitidine in TP53-mutant AML<br>- NCT03931291: Phase II trial of eprenetapopt plus azacitidine as post-transplant maintenance in TP53-mutant AML/MDS<br>[265 - 267]
TUBB2B	N/A	- He 2025 using siRNA-AuNP preclinically<br>[78]
Wnt-3 / Wnt-4	N/A	- NCT02675946, NCT03507998: Phase I/Ib CGX1321 ± pembrolizumab in GI tumors<br>- Phase I LGK-974 in solid tumors<br>- Phase I ETC-159 in advanced solid tumors<br>[268]
GSK-3 (Glycogen Synthase Kinase-3)	N/A	- NCT03678883: Phase I/II trial of elraglusib (9-ING-41) in advanced solid tumors<br>- Phase I/II elraglusib + gemcitabine/nab-paclitaxel in pancreatic cancer<br>- Phase I/II elraglusib in combination with checkpoint inhibitors<br>[269, 270]
αB-crystallin	N/A	-NCT02442570: Phase 2a, DC-TAB (recombinant human alpha B-crystallin) in multiple sclerosis

NLRP3 infammasome + IL-1β	- Canakinumab (Ilaris): human mAb, neutralizes Il-1b<br>- Rilonacept: soluble Il-1a/IL-1b trap<br>- Anakinra: recombinant IL-1 receptor antagonist	- NCT06129409: MT0796, Phase 1/2, inhibits-NLRP3-driven diseases and is BBB penetrant<br>- NCT06822517: VENT-02, NLRP3 inhibitor, Phase 1b in Parkinson's Disease<br>[271 - 273]
